# Treatment strategies for motor fluctuations in Parkinson’s disease: a systematic review of efficacy, functionality, and drug accessibility with a focus on Latin America

**DOI:** 10.3389/fphar.2025.1725248

**Published:** 2025-12-03

**Authors:** Oscar Arias-Carrión, Emmanuel Ortega-Robles

**Affiliations:** 1 División de Neurociencias, Clínica, Instituto Nacional de Rehabilitación Luis Guillermo Ibarra Ibarra, Mexico City, Mexico; 2 Tecnologico de Monterrey, Escuela de Medicina y Ciencias de la Salud, Mexico City, Mexico

**Keywords:** Parkinson’s disease, motor fluctuations, off-time, dopaminergic therapy, deep brain stimulation, access to care, health disparities

## Abstract

**Background:**

Motor fluctuations represent a major therapeutic challenge in Parkinson’s disease, particularly among individuals on long-term levodopa therapy. Although diverse pharmacological and device-aided strategies are available, their comparative efficacy, functional benefits, and real-world accessibility—especially in low- and middle-income countries (LMICs)—remain inadequately characterised.

**Methodology:**

We conducted a systematic review of randomised controlled trials, comparative effectiveness studies, and high-quality observational cohorts evaluating pharmacological, surgical, and experimental interventions for motor fluctuations in Parkinson’s disease. PubMed and Scopus were searched through 31 May 2025. Eligible studies enrolled adults with idiopathic Parkinson’s disease experiencing motor fluctuations despite levodopa therapy and reported outcomes related to OFF/ON-time duration, functional disability (UPDRS-II), and health-related quality of life (PDQ-39 or EQ-5D). Risk of bias was assessed using validated tools, and the certainty of evidence was graded using the GRADE approach. In parallel, we conducted a comparative analysis of drug accessibility across nine Latin American countries (Argentina, Brazil, Chile, Colombia, Costa Rica, Guatemala, Mexico, Panama, and Peru) and the United States, assessing marketing authorisation status and Purchasing Power Parity (PPP)-adjusted prices per Defined Daily Dose (DDD).

**Results:**

Ninety-two studies met the inclusion criteria. High-certainty evidence supports the efficacy of extended-release levodopa (IPX066), opicapone, pramipexole, rotigotine, and safinamide in reducing OFF-time, although improvements in functional disability and quality of life were modest or inconsistent. Moderate-certainty evidence supports device-aided therapies, including levodopa–carbidopa intestinal gel (LCIG), subcutaneous foslevodopa–foscarbidopa, and continuous apomorphine infusion, which achieved larger effects on OFF-time and functional outcomes. Deep brain stimulation (DBS) of the globus pallidus internus was adequate but limited by cost and availability in LMICs. The Latin American analysis revealed substantial cross-country disparities: the United States had the widest therapeutic portfolio (19 approved drugs) and generally lower PPP-adjusted prices for advanced formulations. In contrast, several newer therapies—such as IPX066, opicapone, istradefylline, and foslevodopa–foscarbidopa—were unavailable in most Latin American markets, and price differentials for controlled-release or add-on therapies were often several-fold higher after PPP adjustment.

**Conclusion:**

While multiple pharmacological and device-based interventions effectively reduce OFF-time in Parkinson’s disease, their real-world impact is constrained by uneven global access and affordability. The Latin American region exemplifies these disparities, with limited regulatory availability, heterogeneous pricing, and insufficient inclusion of novel agents in national formularies. Integrating efficacy evidence with accessibility analyses highlights the need for coordinated regional policies—centred on price regulation, health technology assessment, and equitable funding mechanisms—to ensure that advances in treatment translate into meaningful improvements in function and quality of life for patients with Parkinson’s disease worldwide.

## 1 Introduction

Motor fluctuations are a common and disabling complication of long-term levodopa therapy in Parkinson’s disease (PD), characterised by alternating periods of improved mobility (ON-time) and re-emerging motor symptoms (OFF-time) (Armstrong and Okun, 2020). These fluctuations typically emerge within 5–10 years of initiating treatment and significantly affect quality of life (QoL), functional independence, and caregiver burden (Boura et al., 2025; Pena-Zelayeta et al., 2025). Despite the availability of adjunctive pharmacological and device-aided therapies, managing motor fluctuations remains challenging—particularly in real-world settings and low-resource environments.

Over the past 2 decades, a growing number of therapeutic options have emerged to address motor fluctuations, including extended-release levodopa (e.g., IPX066), catechol-O-methyltransferase (COMT) inhibitors (e.g., opicapone), dopamine agonists (e.g., pramipexole, rotigotine), monoamine oxidase-B (MAO-B) inhibitors (e.g., safinamide, rasagiline), and continuous dopaminergic delivery via intestinal gel or subcutaneous infusion (Armstrong and Okun, 2020). Surgical interventions, such as deep-brain stimulation (DBS), have also demonstrated robust efficacy (Rajamani et al., 2024). However, clinical adoption is often hindered by variable functional outcomes, lack of QoL data, and, crucially, limited access—especially in low- and middle-income countries (LMICs) where even essential dopaminergic agents may be inconsistently available.

Many of the most effective therapies, including IPX066, levodopa–carbidopa intestinal gel (LCIG), foslevodopa–foscarbidopa, and DBS, remain largely inaccessible in LMICs due to high cost, complex delivery infrastructure, and regulatory barriers (Armstrong and Okun, 2020; Li et al., 2025). Even within Latin America, access to key antiparkinsonian medications such as pramipexole, rasagiline, or rotigotine varies widely across countries and between public and private health systems (Dorsey et al., 2018; Kim et al., 2024). These disparities perpetuate a two-tiered system of PD care in which therapeutic decisions are often constrained less by clinical evidence than by economic and geographic inequities.

This systematic review was designed to synthesise high-quality evidence on pharmacological, surgical, and experimental interventions for motor fluctuations in PD, with emphasis on efficacy, functional outcomes, and health-related quality of life. As a secondary objective, it includes a regional analysis of drug accessibility and affordability across Latin American countries, comparing regulatory availability and purchasing-power–adjusted prices with those in the United States. By integrating evidence of therapeutic benefit with real-world data on access, this study aims to provide a comprehensive perspective on both the scientific advances and the persistent inequalities that shape PD management globally, with particular attention to the Latin American context.

## 2 Methods

### 2.1 Search strategy

We conducted a systematic review of pharmacological, surgical, and experimental interventions for motor fluctuations in Parkinson’s disease, focusing on therapeutic efficacy, functional impact, health-related quality of life (HR-QoL), and accessibility in low-resource settings. Eligible studies included randomised controlled trials, high-quality observational studies, and comparative interventional analyses.

Searches were conducted in PubMed (via MEDLINE) and Scopus from inception to 31 May 2025. Search terms combined MeSH and free-text keywords related to “Parkinson’s disease,” “motor fluctuations,” “OFF-time,” “adjunctive therapy,” “device-aided interventions,” and “access to treatment.” Additional terms addressing access barriers included “health disparities,” “low- and middle-income countries,” and “availability of Parkinson’s therapies.”

The studies included in the analysis met the following criteria: participants had established motor fluctuations despite levodopa therapy; the interventions evaluated were pharmacological, surgical, or device-aided strategies aimed at reducing OFF-time or increasing ON-time; the outcomes reported included measures of motor fluctuations, functional status (such as the second part of the Unified Parkinson’s Disease Rating Scale, UPDRS-II), or health-related quality of life (such as Parkinson’s Disease Questionnaire, PDQ-39 or EuroQol 5 Dimension, EQ-5D); and each study incorporated a comparison group. The minimum required follow-up period was 12 weeks for pharmacological treatments and 6 months for surgical interventions.

Two reviewers independently screened titles and abstracts; after that, the full texts of eligible studies were reviewed, and relevant data were extracted. This review was conducted and reported in accordance with the PRISMA 2020 guidelines. The complete search strategy, PRISMA flow diagram, and supporting tables are provided in the Supplementary Information. No formal protocol was registered for this review. The certainty of evidence supporting each pharmacological, surgical, or experimental treatment was graded as high, moderate, low, or very low using the GRADE (Grading of Recommendations Assessment, Development and Evaluation) framework. In this approach, randomised controlled trials were initially considered high-certainty evidence, and observational studies were considered low-certainty evidence. The rating could then be downgraded or upgraded based on standardised GRADE criteria, including risk of bias, inconsistency, indirectness, imprecision, and publication bias.

### 2.2 Data analysis

We synthesised data from 92 eligible studies to evaluate and score each pharmacological treatment across four key domains: efficacy, functional benefit, health-related quality of life (HR-QoL) impact, and availability in low- and middle-income countries (LMICs). For each domain, treatments were assigned a semi-quantitative score ranging from 0 (poor or absent) to 3 (excellent). These ratings were not intended as precise quantitative measurements but rather as comparative indicators summarising the relative strength of the evidence and clinical relevance observed across studies. Efficacy scores were based on the overall magnitude and consistency of OFF-time reduction reported in the studies. Improvements in disability scales, such as the UPDRS-II, were assessed for functional benefit. The HR-QoL domain assessed the presence and extent of clinically meaningful changes in instruments such as the PDQ-39 or EQ-5D. Availability was evaluated by examining inclusion in public formularies and essential medicines lists, as well as accessibility information obtained from the World Health Organisation (WHO) regional reports and national regulatory databases. This scoring approach involved some subjective interpretation by the authors, representing a qualitative synthesis of heterogeneous evidence rather than a statistical estimate. Importantly, this semi-quantitative scoring system is conceptually distinct from the GRADE framework, which was separately applied to assess the certainty of the evidence supporting each treatment.

All evaluations were performed independently by two reviewers, and any discrepancies were resolved by consensus. The resulting scores were integrated into a matrix and visualised as a heatmap in R (version 4.5.1) using the ggplot2 package, with colour intensity representing the relative performance of each treatment across domains. Treatments were listed by their generic or formulation names and stratified by the certainty of supporting evidence.

### 2.3 Analysis of drug accessibility in the Latin American region

To assess the accessibility of antiparkinsonian medications across Latin America, we selected nine representative countries: Mexico, Argentina, Brazil, Chile, Colombia, Peru, Guatemala, Costa Rica, and Panama. This selection was designed to ensure both regional and economic representativeness, encompassing all major subregions of Latin America—North America, South America, Central America, and the Caribbean. The group includes the region’s largest pharmaceutical markets, such as Brazil, Mexico, and Argentina, as well as mid-sized and emerging markets like Chile and Colombia, and smaller Central American economies such as Costa Rica, Guatemala, and Panama. Countries were also selected based on the availability of reliable regulatory information through official national agencies, facilitating verification of drug registration and market authorisation. Additionally, prices in the United States were retrieved for reference, enabling direct comparison with those in the Latin American region.

First, we verified which antiparkinsonian drugs were authorised for commercial distribution (i.e., had a valid marketing authorisation or *registro sanitario*, as it is called in most Latin American countries). For this purpose, each drug was searched in the official databases of the national regulatory agencies of the selected countries (see Supplementary Table S1 for the complete list of agencies and their websites). However, the regulatory agency website of Panama was not operational at the time of data collection, so this information could not be retrieved.

Subsequently, drugs with confirmed registration were searched for on at least 3 online pharmacies in each country. Pharmacies were chosen based on their national distribution, reliability, and the breadth of their online catalogues (see Supplementary Information for the specific pharmacies and URLs). When available, official reference prices published by national regulatory agencies (Argentina, Brazil, and the United States) were also considered. Only products with identifiable dosage strength and package size information were included; products lacking unit price data were excluded. Because the same drug is marketed under different brands, dosages, and package sizes across countries, all available presentations (generic and branded) were extracted from each pharmacy’s catalogue. Prices were then averaged per presentation and converted from the local currency to U.S. dollars using the average monthly exchange rate for June 2025, as reported by the International Monetary Fund (IMF). Prices were subsequently expressed as cost per Defined Daily Dose (DDD), and the mean DDD-based price was calculated across all available presentations. DDD values for each medication were obtained from the WHO Collaborating Centre for Drug Statistics Methodology. To account for differences in purchasing power across countries, all prices were further adjusted for Purchasing Power Parity (PPP) using the 2024 annual averages reported by the World Bank, expressed relative to the United States. In Panama, due to national pharmaceutical regulations, pharmacies do not publicly disclose prices for this class of medications. Some price data for this country were obtained through direct consultation with a local pharmacy; however, this information may be incomplete and not fully representative of national pricing. We nonetheless decided to retain Panama in the analysis to preserve regional representativeness within Central America and to avoid potential selection bias related to economic or regulatory heterogeneity.

Specific considerations were applied during both regulatory and pricing searches. Pramipexole was treated separately for its immediate-release (IR) and extended-release (ER) formulations. Amantadine was analysed only as monotherapy and in its immediate-release form, as this is the most widely marketed across countries. Entacapone was included only as a single-agent formulation, excluding fixed-dose combinations with levodopa. Nicotine was considered exclusively in its transdermal patch presentation. Additional assumptions were made for DDD calculations. Since the WHO does not distinguish between IR and ER formulations of pramipexole, a 1:1 conversion factor was applied, following the equivalence reported by Rascol et al. (2010). As no specific DDD is defined for IPX066, the DDD of oral levodopa–carbidopa was used as reference and converted according to Tetrud et al. (2017). For LCIG, a conversion factor of 1 was applied based on the FDA’s Duopa™ labelling and Pedersen et al. (2012). For foslevodopa–foscarbidopa, a 1.3 conversion factor was applied as recommended in the FDA’s Vyalev™ labelling, and for levodopa–carbidopa controlled-release (CR), a 1:1 conversion was used based on the FDA’s Sinemet CR™ labelling. As istradefylline lacks an official DDD, we used the FDA-recommended daily dose from the Nourianz™ labelling. Terguride was not included in the comparison because no DDD has been established and the drug is currently marketed only in Japan.

All data were collected between July and August 2025 to ensure temporal consistency across sources. Data analysis and graphical representations were performed using Microsoft Excel 365 (Microsoft Corp., United States) and RStudio (version 2025.09.1) running R (version 4.5.1).

## 3 Results

Motor fluctuations in PD reflect both the limitations of chronic levodopa therapy and the challenges of sustaining functional independence over time. This review synthesised evidence from 92 studies evaluating pharmacological, surgical, and experimental strategies to mitigate OFF-time, improve daily function, and enhance HR-QoL. Treatments were categorised by evidence certainty and contextualized by their real-world availability, especially in LMICs, where access disparities often shape therapeutic decision-making.

**FIGURE 1:**
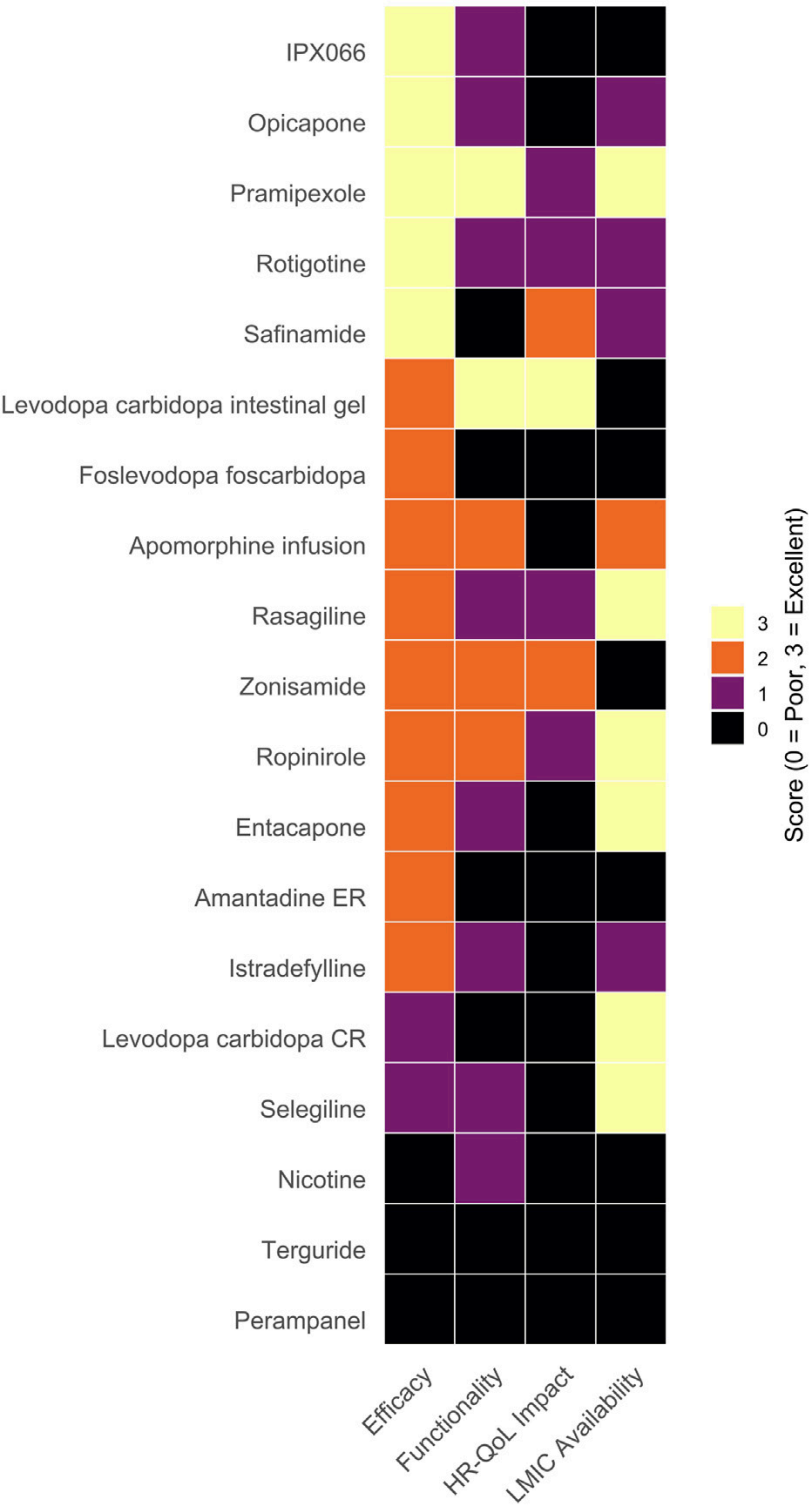
Comparative semi-quantitative assessment of pharmacological treatments for motor fluctuations in Parkinson’s disease. Each treatment was evaluated across four domains—efficacy, functional benefit, health-related quality of life (HR-QoL) impact, and availability in low- and middle-income countries (LMICs)—using a semi-quantitative ordinal scale from 0 (poor or absent) to 3 (excellent). Scores were assigned based on a qualitative synthesis of the available evidence: efficacy was judged according to the magnitude and quality of OFF-time reduction; functional benefit according to improvements in disability scales such as the UPDRS-II; HR-QoL impact based on clinically meaningful changes in instruments such as the PDQ-39 or EQ-5D; and LMIC availability according to inclusion in essential medicines lists and national formularies. Ratings were determined independently by two reviewers and reflect the strength and consistency of evidence from randomised controlled trials and high-quality observational studies. Colour intensity represents performance across domains, with lighter tones indicating higher scores. Overall, high-efficacy agents (e.g., IPX066, opicapone, pramipexole, rotigotine) show variable functional or HR-QoL impact and inconsistent availability in LMICs.

**FIGURE 2:**
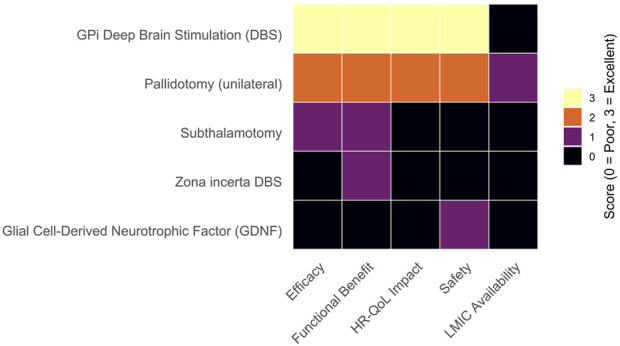
Comparative semi-quantitative assessment of surgical and experimental interventions for motor fluctuations in Parkinson’s disease. Each procedure was evaluated across five domains—efficacy, functional benefit, health-related quality of life (HR-QoL) impact, safety, and availability in low- and middle-income countries (LMICs)—using a semi-quantitative ordinal scale from 0 (poor or absent) to 3 (excellent). Scores were assigned based on a qualitative synthesis of the available clinical evidence, considering the magnitude and consistency of motor and functional improvements (e.g., UPDRS-II/III changes), the extent of HR-QoL gains, the frequency and severity of adverse or cognitive effects, and the practical accessibility of each procedure in LMIC settings. Ratings were determined independently by two reviewers and reflect the strength and clinical relevance of the evidence from randomised controlled trials and high-quality observational studies. Colour intensity represents performance across domains, with lighter tones indicating higher scores. Internal globus pallidus deep brain stimulation (GPi-DBS) provides the most substantial evidence of efficacy and functional benefit but remains inaccessible mainly in LMICs. Pallidotomy provides moderate benefits with broader feasibility, while other interventions show limited, heterogeneous, or emerging evidence, often constrained by safety or implementation challenges.

Across pharmacological interventions, high-certainty evidence confirms that several adjunctive therapies effectively reduce OFF-time; however, their effect on functional disability and HR-QoL is often modest or inconsistently reported. Moderately supported device-aided therapies showed more robust functional improvements but remain inaccessible in most LMICs. Surgical approaches—particularly globus pallidus deep brain stimulation (GPi-DBS)—demonstrated substantial motor and QoL benefits, but are similarly constrained by infrastructure and cost. A visual synthesis of efficacy, functional outcomes, and accessibility is presented in Figures 1, 2, accompanied by detailed evidence stratification in Tables 1, 2.

**TABLE 1:** Pharmacological treatments for motor fluctuations in Parkinson’s disease.

Treatment	Efficacy	Functional benefit	HR-QoL impact	Availability in LMICs	References
High-quality evidence					
IPX066 (extended-release)	High	Modest (below MCID)	Not clinically relevant	Rarely available	Hauser et al. (2013)
Opicapone	High	Minimal	No significant benefit	Not widely available	Ferreira et al. (2016), Lees et al. (2017), Takeda et al. (2021)
Pramipexole (IR/ER)	High	Consistent improvement	Variable	Widely available	Mizuno et al. (2003), Wong et al. (2003), Moller et al. (2005), Poewe et al. (2007), Schapira et al. (2011), Mizuno et al. (2012)
Rotigotine	High	Variable	Limited data	Not widely available	LeWitt et al. (2007), Poewe et al. (2007), Mizuno et al. (2014), Nicholas et al. (2014), Nomoto et al. (2014), Zhang et al. (2017)
Safinamide	High	Not evaluated	Benefit in 1/4 trials	Limited access	Borgohain et al. (2014), Schapira et al. (2017), Hattori et al. (2020b), Wei et al. (2022)
Moderate-quality evidence					
Levodopa–carbidopa intestinal gel	Moderate	Clinically relevant	Improved (PDQ-39)	Not available	Olanow et al. (2014), Chung et al. (2022)
Foslevodopa–foscarbidopa (subcutaneous)	Moderate	Not assessed	No data	Not available	Soileau et al. (2022)
Apomorphine infusion	Moderate	Modest	No benefit	Available in select centres	Hattori et al. (2014), Katzenschlager et al. (2018), Olanow et al. (2020)
Rasagiline	Moderate	Small benefit	Minimal	Widely available	Parkinson Study Group (2005), Rascol et al. (2005), Zhang et al. (2013a), Hattori et al. (2018), Zhang et al. (2018)
Zonisamide	Moderate	Modest	Not evaluated	Regionally available (e.g., Japan)	Murata et al. (2007), Murata et al. (2015)
Ropinirole (IR)	Moderate	Modest	Inconsistent	Widely available	Rascol et al. (1996), Lieberman et al. (1998), Brunt et al. (2002), Im et al. (2003), Barone et al. (2007), Mizuno et al. (2007), Pahwa et al. (2007), Watts et al. (2010), Reichmann et al. (2011), Stocchi et al. (2011), Zhang et al. (2013b), Mizuno et al. (2014), Zesiewicz et al. (2017), Hattori et al. (2020a)
Entacapone	Moderate	Very low evidence for benefit	Insufficient	Widely available	Parkinson Study Group (1997), Rinne et al. (1998), Poewe et al. (2002), Brooks et al. (2003), Fenelon et al. (2003), Reichmann et al. (2005), Deuschl et al. (2007), Lew et al. (2011)
Amantadine ER	Moderate	Not evaluated	Not assessed	Rarely available	Ory-Magne et al. (2014), Oertel et al. (2017), Pahwa et al. (2017)
Istradefylline	Moderate	Limited	Not assessed	Not widely available	Hauser et al. (2003), Hauser et al. (2008), LeWitt et al. (2008), Stacy et al. (2008), Mizuno et al. (2010), Pourcher et al. (2012), Mizuno et al. (2013), Li et al. (2015)
Low-quality or insufficient evidence					
Levodopa–carbidopa CR	Low	No improvement	No effect	Widely available	Hutton et al. (1988), Wolters et al. (1992), Wolters and Tesselaar (1996)
Selegiline	Low	Inconsistent	No benefit	Widely available	Heinonen et al. (1989), Teychenne and Parker (1989), Hubble et al. (1993), Waters et al. (2004), Ondo et al. (2007)
Nicotine (patch)	Very low	Unblinded improvement	Not assessed	Not used for PD	Villafane et al. (2018)
Terguride	Very low	No effect	Not assessed	Not available	Pacchetti et al. (1993)
Perampanel	Very low	No benefit	Not assessed	Not available	Eggert et al. (2010), Lees et al. (2012)

**TABLE 2:** Surgical and experimental treatments for motor fluctuations in Parkinson’s disease.

Procedure	Efficacy	Functional benefit	HR-QoL impact	Safety	Availability in LMICs	References
High-quality evidence						
GPi Deep Brain Stimulation (DBS)	High	UPDRS-II and III improved	PDQ-39 improved	Good cognitive profile	Limited to tertiary centres	Anderson et al. (2005), Weaver et al. (2009), Follett et al. (2010), Williams et al. (2010), Weaver et al. (2012), Odekerken et al. (2013), Sidiropoulos et al. (2016)
Moderate-quality evidence						
Pallidotomy (unilateral)	Moderate	Sustained motor benefit	Modest improvement	No sham controls; risks lower than subthalamotomy	Available in some LMICs	de Bie et al. (1999), Vitek et al. (2003), Esselink et al. (2004), Esselink et al. (2006), Coban et al. (2009)
Insufficient or conflicting evidence						
Subthalamotomy	Low	Modest benefit	Not assessed	High hemiballismus rates (20%–50%)	Rarely performed	Merello et al. (2008), Coban et al. (2009)
Zona incerta DBS	Very low	Some motor benefit	No group difference	High risk of bias	Not available	Blomstedt et al. (2018)
Glial Cell-Derived Neurotrophic Factor (GDNF)	Very low	No clinical effect	No improvement	Safe but ineffective	Experimental	Whone et al. (2019a), Whone et al. (2019b)

The following sections present disaggregated findings for each intervention, grouped by evidence quality and treatment modality.

### 3.1 Pharmacological treatment for motor fluctuations in Parkinson’s disease

#### 3.1.1 High-quality evidence

##### 3.1.1.1 IPX066 (levodopa–carbidopa extended release)

IPX066 is efficacious in reducing motor fluctuations in individuals with PD on (attempted) optimized oral levodopa therapy. A single, high-quality RCT evaluated IPX066 against levodopa–carbidopa IR in patients with established motor fluctuations (Hauser et al., 2013). IPX066 significantly reduced OFF-time by a mean of 1.2 h per day (P < 0.0001), accompanied by increases in ON-time without troublesome dyskinesia (0.9 h; P < 0.001) and ON-time without any dyskinesia (0.7 h; P < 0.05). These changes surpassed the established minimal clinically important difference (MCID) and were consistent with clinically meaningful benefits.

Additionally, dosing frequency was reduced with IPX066 (mean 3.6 vs. 5.0 daily doses; P < 0.0001), potentially improving adherence and treatment satisfaction. However, improvements in disability were modest and fell below the MCID: UPDRS-II scores improved by 0.9 points during the ON state and 0.8 points during the OFF state (both P < 0.005), but these changes were not clinically relevant. Similarly, quality-of-life outcomes did not show a significant benefit, with a PDQ-39 score difference of 2.5 points (P < 0.05), which did not meet the threshold for clinical importance. Other measures (e.g., SF-36, EQ-5D) showed no significant differences between groups.

While IPX066 demonstrates efficacy in managing motor fluctuations with reduced dosing burden, its influence on functional disability and quality of life remains limited. These findings underscore the need for future trials that integrate patient-centred outcomes and real-world effectiveness. Additional head-to-head studies comparing extended-release formulations are warranted to guide individualised treatment decisions based on fluctuation patterns, treatment adherence, and patient preferences.

##### 3.1.1.2 Opicapone

Opicapone is efficacious in the treatment of motor fluctuations in people with PD receiving (attempted) optimized oral levodopa therapy. Three high-quality placebo-controlled trials evaluated the effect of once-daily opicapone in patients with motor fluctuations (Ferreira et al., 2016; Lees et al., 2017; Takeda et al., 2021). Two trials reported a clinically relevant reduction in OFF-time of approximately 120 min per day, along with increases in ON-time exceeding 60 min compared with placebo (Ferreira et al., 2016; Lees et al., 2017). The third study observed a smaller benefit, with an OFF-time reduction of approximately 0.7 h (Takeda et al., 2021).

Despite consistent motor benefit, none of the three trials showed statistically or clinically significant improvements in QoL, as assessed by the PDQ-39. Disability outcomes were assessed in two trials. One study reported no improvement (Lees et al., 2017), while another found a small but statistically significant difference (approximately 1 point in the UPDRS-II) that did not meet clinical significance thresholds (Takeda et al., 2021). Opicapone offers a convenient, once-daily treatment with consistent efficacy in OFF-time reduction. However, its weak effect on disability and HR-QoL highlights the need for more comprehensive outcome measures in future trials.

##### 3.1.1.3 Pramipexole

Both immediate-release and extended-release formulations of pramipexole are efficacious in managing motor fluctuations in people with PD receiving (attempted) optimized levodopa therapy. Six trials fulfilled the inclusion criteria (Mizuno et al., 2003; Wong et al., 2003; Moller et al., 2005; Poewe et al., 2007; Schapira et al., 2011; Mizuno et al., 2012). Five placebo-controlled trials have consistently demonstrated that pramipexole reduces OFF-time to a clinically relevant extent (Wong et al., 2003; Poewe et al., 2007; Schapira et al., 2011) and improves MDS-UPDRS-II scores during the off-medication state (Mizuno et al., 2003; Moller et al., 2005; Schapira et al., 2011). One study also showed significant improvement in ON-time without troublesome dyskinesia and in HR-QoL (PDQ-39) (Poewe et al., 2007), although this was not confirmed in all trials (Schapira et al., 2011).

Two studies compared extended- and immediate-release formulations with placebo and reported comparable improvements in OFF-time and MDS-UPDRS-II scores (Poewe et al., 2007; Schapira et al., 2011), with one trial also reporting benefit on the PDQ-39 total score (Mizuno et al., 2012). Active-comparator trials found noninferiority of pramipexole compared with both rotigotine (Poewe et al., 2007) and bromocriptine (Mizuno et al., 2003), reinforcing its clinical utility. These findings collectively support the efficacy of pramipexole, with meaningful benefits in motor symptom control and functional capacity. QoL improvements, while observed in some trials, remain variable and warrant further investigation.

##### 3.1.1.4 Rotigotine

Rotigotine is efficacious in reducing motor fluctuations in people with PD receiving (attempted) optimized oral levodopa therapy. Six placebo-controlled trials met the inclusion criteria for evaluating rotigotine (LeWitt et al., 2007; Poewe et al., 2007; Mizuno et al., 2014; Nomoto et al., 2014; Zhang et al., 2017). All reported statistically significant reductions in OFF-time compared with placebo, with five of the six studies demonstrating a treatment effect exceeding 1 h per day—considered clinically significant (LeWitt et al., 2007; Poewe et al., 2007; Mizuno et al., 2014; Nicholas et al., 2014; Nomoto et al., 2014; Zhang et al., 2017). The sixth study, although showing a treatment effect of 0.9 h per day, was confounded by an unusually large placebo response (1.5 h per day OFF-time reduction in the placebo arm) (Nicholas et al., 2014).

Regarding functional disability, all six trials reported numerical improvements, with four reaching statistical significance (LeWitt et al., 2007; Poewe et al., 2007; Mizuno et al., 2014; Nomoto et al., 2014). However, two studies, including one with a large sample size (n = 406), did not demonstrate statistically significant changes in disability scores (Nicholas et al., 2014; Zhang et al., 2017). HR-QoL was assessed in two studies using the PDQ-39 (Poewe et al., 2007) and PDQ-8 (Zhang et al., 2017), with statistically significant improvement observed in only one (Poewe et al., 2007). Thus, although rotigotine consistently reduces motor fluctuations, evidence for improvements in disability and HR-QoL is weak. These results confirm rotigotine as a clinically effective option for managing motor fluctuations; however, additional studies are required to clarify its role in improving daily function and quality of life across diverse PD populations.

##### 3.1.1.5 Safinamide

Safinamide is efficacious in reducing motor fluctuations in people with PD receiving (attempted) optimized oral levodopa therapy. Four placebo-controlled trials evaluated safinamide at doses of 50 mg and 100 mg per day (Borgohain et al., 2014; Schapira et al., 2017; Hattori et al., 2020b; Wei et al., 2022). All studies reported a statistically and clinically important reduction in OFF-time of approximately 1 h per day compared with placebo, along with a corresponding increase in ON-time without troublesome dyskinesia.

However, none of the trials assessed motor impairment using UPDRS-III or MDS-UPDRS-III in the off-medication state. Regarding QoL, outcomes were mixed. Only one study met the 5-point threshold for clinical relevance on the PDQ-39, though the wide confidence interval introduced uncertainty (Borgohain et al., 2014). In one study, only the 100 mg dose achieved a clinically meaningful improvement (Hattori et al., 2020b), whereas two other studies failed to reach this threshold (Schapira et al., 2017; Hattori et al., 2020b).

Although functional and QoL data are limited, the consistent benefit in OFF and ON time control supports the use of safinamide as an effective adjunct to levodopa in patients with motor fluctuations. Further research should address long-term disability outcomes and patient-centred measures.

#### 3.1.2 Moderate-quality evidence

##### 3.1.2.1 Levodopa–carbidopa intestinal gel

Levodopa–carbidopa intestinal gel is likely efficacious for the treatment of motor fluctuations in people with PD receiving (attempted) optimised oral levodopa therapy. Two trials met the eligibility criteria (Olanow et al., 2014; Chung et al., 2022). One double-blind, placebo-controlled trial evaluated 16-h/day LCIG infusion and reported a reduction in OFF-time of 1.9 h per day (P < 0.01) compared with levodopa–carbidopa immediate-release (Olanow et al., 2014). The same study showed increases in ON-time without troublesome dyskinesia (1.9 h; P < 0.01) and ON-time without any dyskinesia (2.3 h; P = 0.01), as well as improvements in disability (UPDRS-II: 3-point reduction; P < 0.001) and HR-QoL (PDQ-39: 7-point improvement; P < 0.05), both exceeding minimal clinically important differences.

A second, open-label comparison against best medical therapy (BMT) demonstrated greater improvement in disability with the intestinal gel (UPDRS-II: 2.3 vs. 0.5 points; P = 0.006) (Chung et al., 2022). However, this trial did not show meaningful differences in motor impairment or HR-QoL. While findings are promising, they are based on a relatively small dataset and raise concerns regarding the risk of bias and short follow-up (3 months). The current evidence suggests a likely clinical benefit, but additional long-term and head-to-head comparative data are needed to confirm its efficacy and establish its position within treatment algorithms.

##### 3.1.2.2 Continuous subcutaneous foslevodopa–foscarbidopa

Continuous subcutaneous foslevodopa–foscarbidopa is likely efficacious for the treatment of motor fluctuations in people with PD receiving (attempted) optimised oral levodopa therapy—a single placebo-controlled trial assessed 24-h continuous subcutaneous delivery of foslevodopa–foscarbidopa (Soileau et al., 2022). The intervention reduced OFF-time by 1.8 h per day (P = 0.002) and increased ON-time without troublesome dyskinesia by 1.8 h (P < 0.01) compared with levodopa–carbidopa immediate release. However, the trial did not report clinically significant changes in disability or HR-QoL.

The lack of data on functional outcomes and quality of life, coupled with reliance on a single study, limits the strength of the evidence. Nevertheless, the consistency of motor symptom control suggests potential benefits, particularly for patients requiring continuous dopaminergic delivery. Future studies should focus on durability, adverse effects, and the impact on non-motor symptoms.

##### 3.1.2.3 Apomorphine

Continuous subcutaneous apomorphine infusion is likely efficacious for the treatment of motor fluctuations in people with PD receiving (attempted) optimised oral levodopa therapy. The evidence is insufficient to substantiate the efficacy of intermittent apomorphine formulations—such as sublingual film or subcutaneous pen—when used as rescue therapy for off periods. Three trials fulfilled the inclusion criteria (Hattori et al., 2014; Katzenschlager et al., 2018; Olanow et al., 2020). One high-quality, placebo-controlled study evaluated continuous subcutaneous apomorphine infusion administered for approximately 16 h daily (Katzenschlager et al., 2018). It demonstrated a clinically relevant reduction in OFF-time of 1.9 h per day (P = 0.0025) and an increase in ON-time without troublesome dyskinesia of 2.0 h (P = 0.0008). However, the trial had limitations, including potential unblinding due to skin nodules at infusion sites.

Apomorphine sublingual film (Olanow et al., 2020) and intermittent subcutaneous injection (Hattori et al., 2014) were evaluated as rescue therapies during off episodes. Both achieved rapid improvement in motor symptoms, transitioning patients from OFF to ON within 15–40 min, as assessed by the MDS-UPDRS-III. However, neither intervention led to sustained changes in disability (MDS-UPDRS-II) or quality of life (PDQ-8 or PDQ-39) over 12 weeks. Thus, while continuous subcutaneous infusion appears promising for managing chronic motor fluctuations, the current evidence base is limited to a single RCT. Further long-term, head-to-head studies are needed to assess comparative efficacy, safety, and patient-reported outcomes. The role of intermittent apomorphine remains uncertain due to the lack of durable functional or QoL benefits.

##### 3.1.2.4 Rasagiline

Rasagiline is likely efficacious in the treatment of motor fluctuations in people with PD on (attempted) optimized oral levodopa therapy. Five placebo-controlled trials evaluated rasagiline in patients experiencing motor fluctuations (Parkinson Study Group, 2005; Rascol et al., 2005; Zhang L. et al., 2013; Hattori et al., 2018; Zhang et al., 2018). Across all studies, rasagiline reduced OFF-time by 0.5–0.9 h per day. While these reductions were statistically significant, they fell short of the minimal clinically important difference, leading to a conclusion of likely efficacy.

Motor symptom improvement (as assessed by UPDRS-III or MDS-UPDRS-III in the off-medication state) and disability (as assessed by UPDRS-II or MDS-UPDRS-II) were evaluated in four studies (Parkinson Study Group, 2005; Rascol et al., 2005; Zhang L. et al., 2013; Hattori et al., 2018). All reported statistically significant improvements; however, the magnitude of change (1.6–5.6 points for motor scores and 1.0 to 1.7 points for disability) did not reach clinical relevance. Increases in ON-time without troublesome dyskinesia ranged from 0.5 to 1.2 h, again below the threshold for clinical significance. Three studies evaluated HR-QoL using the PDQ-39 (Parkinson Study Group, 2005; Hattori et al., 2018; Zhang et al., 2018), reporting small but statistically significant improvements that were not clinically meaningful. One trial reported a dose-dependent increase in dyskinesia, suggesting a narrow therapeutic window for higher doses. Overall, rasagiline provides modest but consistent benefits for motor symptoms and ON-time. Its limited effect on disability and HR-QoL, combined with potential side effects at higher doses, suggests a role as adjunctive therapy in selected patients rather than a first-line option for motor fluctuation management.

##### 3.1.2.5 Zonisamide

Zonisamide is likely efficacious in reducing OFF-time in people with PD and motor fluctuations on (attempted) optimised oral levodopa therapy. Two placebo-controlled trials conducted by the same research group investigated zonisamide at daily doses of 25, 50, and 100 mg (Murata et al., 2007; Murata et al., 2015). OFF-time was reduced by 40–85 min, demonstrating a dose-dependent effect. Improvements in UPDRS-III (on-medication state) ranged from 1.5 to 3.8 points compared with placebo, but between-trial inconsistencies and high dropout rates (approximately 20%) reduced overall confidence.

One study evaluated disability (UPDRS-II) and found a statistically significant but clinically modest 1-point improvement in the off-medication state (Murata et al., 2015). Changes in ON-time and HR-QoL were not assessed. Although zonisamide shows promise for reducing OFF episodes, additional independent studies are needed to validate its efficacy and explore functional and quality-of-life outcomes.

##### 3.1.2.6 Ropinirole

Ropinirole immediate-release is efficacious, whereas ropinirole prolonged-release and transdermal patch formulations are likely efficacious for treating motor fluctuations in individuals with PD who are receiving oral levodopa therapy. Fourteen RCTs were included in the analysis (Rascol et al., 1996; Lieberman et al., 1998; Brunt et al., 2002; Im et al., 2003; Barone et al., 2007; Mizuno et al., 2007; Pahwa et al., 2007; Watts et al., 2010; Reichmann et al., 2011; Stocchi et al., 2011; Zhang Z. et al., 2013; Mizuno et al., 2014; Zesiewicz et al., 2017; Hattori et al., 2020a). Four trials demonstrated that immediate-release ropinirole reduces OFF-time compared with placebo, with changes approaching or achieving clinical relevance (Rascol et al., 1996; Lieberman et al., 1998; Barone et al., 2007; Mizuno et al., 2007). However, improvements in ON-time, disability, and HR-QoL were either modest or non-significant. Three trials investigating ropinirole prolonged-release formulations reported reduced OFF-time; however, due to the high risk of bias in two of these trials, the conclusion was downgraded to likely efficacious (Pahwa et al., 2007; Zhang L. et al., 2013; Zesiewicz et al., 2017). ON-time improvements were consistent across studies, but disability outcomes showed minimal change. One study on a ropinirole transdermal patch demonstrated a clinically relevant reduction in OFF-time compared with a placebo patch, but no differences in ON-time or functional disability (Hattori et al., 2020a).

A head-to-head trial comparing immediate- and prolonged-release formulations reported no meaningful differences in OFF-time, ON-time, or HR-QoL, providing insufficient evidence to support the superiority of one formulation over the other (Stocchi et al., 2011). Additional trials comparing ropinirole with rotigotine, bromocriptine, and adjunctive levodopa therapy have found comparable efficacy in reducing OFF-time and achieving functional outcomes (Brunt et al., 2002; Im et al., 2003; Watts et al., 2010; Mizuno et al., 2014). Although immediate-release ropinirole offers consistent efficacy in reducing motor fluctuations, the extended-release and patch formulations may improve adherence and reduce dosing frequency. However, their efficacy is less robustly supported and demands further investigation. Overall, ropinirole remains a valuable adjunctive therapy, though the degree of benefit for disability and HR-QoL remains uncertain due to limited supporting evidence.

##### 3.1.2.7 Entacapone

Entacapone is likely efficacious in reducing motor fluctuations in people with PD compared with a placebo. However, there is currently no convincing evidence to support its superiority over active comparators, such as cabergoline, or to determine the added value of an immediate switch to levodopa–carbidopa–entacapone compared with a delayed switch. Six placebo-controlled trials investigated the use of entacapone as an adjunct to levodopa in patients with motor fluctuations (Parkinson Study Group, 1997; Rinne et al., 1998; Poewe et al., 2002; Brooks et al., 2003; Fenelon et al., 2003; Reichmann et al., 2005). All trials assessed ON-time and OFF-time duration, with five reporting reductions in OFF-time that approached or met clinical relevance. However, moderate concerns about allocation concealment and methodological variability led to a downgraded level of evidence. Disability outcomes were evaluated across all six trials, but due to inconsistencies, imprecision, and unclear allocation concealment, the strength of the evidence supporting a benefit in functional outcomes was rated as very low. The quality of life was assessed in only two studies (Parkinson Study Group, 1997; Fenelon et al., 2003), both with similar concerns about bias and precision, yielding inconclusive evidence for this outcome.

Two additional trials compared entacapone to active controls. One small study found no superiority of entacapone over cabergoline, and the lack of replication or power precludes firm conclusions (Deuschl et al., 2007). Another open-label study investigated an immediate versus delayed switch to levodopa–carbidopa–entacapone and assessed HR-QoL at 16 weeks. Due to the open-label design, risk of bias, and imprecision, evidence from this study was also deemed insufficient (Lew et al., 2011). In summary, entacapone may offer modest benefits in reducing OFF-time but lacks strong evidence for improving disability or HR-QoL, and its comparative efficacy remains uncertain.

##### 3.1.2.8 Amantadine

Amantadine extended-release is likely efficacious in increasing ON-time and reducing OFF-time in people with PD and motor fluctuations on (attempted) optimised oral levodopa therapy. Current findings do not provide consistent support for the efficacy of immediate-release amantadine. Two placebo-controlled trials assessed amantadine extended-release as an adjunct therapy in patients with levodopa-induced motor fluctuations (Oertel et al., 2017; Pahwa et al., 2017). Both studies reported improvements of clinical relevance in ON-time, including increases in ON-time without troublesome dyskinesia, with troublesome dyskinesia, and total ON-time. Although reductions in OFF-time did not meet clinical relevance thresholds, the magnitude of the effect indicated a significant treatment benefit.

A third study evaluated amantadine withdrawal and found that discontinuation significantly worsened dyskinesia and reduced ON-time without a concomitant increase in OFF-time (Ory-Magne et al., 2014). None of the trials assessed motor impairment (MDS-UPDRS-III) or disability (MDS-UPDRS-II) in the off-medication state, and quality-of-life outcomes were not reported.

These findings suggest that extended-release amantadine provides a meaningful benefit in managing dyskinesia and enhancing ON-Time Adherence; however, its role in disability and HR-QoL remains undefined. Evidence for immediate-release formulations is currently insufficient.

##### 3.1.2.9 Istradefylline

Istradefylline is likely efficacious for the treatment of motor fluctuations in people with PD on (attempted) optimized oral levodopa therapy. Eight trials evaluated istradefylline at daily doses ranging from 10 mg to 60 mg (Hauser et al., 2003; Hauser et al., 2008; LeWitt et al., 2008; Stacy et al., 2008; Mizuno et al., 2010; Pourcher et al., 2012; Mizuno et al., 2013; Li et al., 2015). One trial also assessed istradefylline combined with sham repetitive transcranial magnetic stimulation (rTMS) (Li et al., 2015). OFF-time reduction varied across studies, ranging from as little as 12 min in larger trials to up to 114 min in smaller trials. Similar variability was observed in ON-time without troublesome dyskinesia, with increases ranging from 12 to 48 min, below the threshold for clinical relevance in most studies.

Notably, several trials reported an increase in ON-Time Episodes with troublesome dyskinesia, ranging from 6 to 60 min (LeWitt et al., 2008; Stacy et al., 2008; Mizuno et al., 2010; Mizuno et al., 2013), which may limit tolerability in some patients.

There were no significant improvements in motor impairment scores (UPDRS-III) in either the ON- or off-medication states. Reported changes ranged from −2.5 to 1.4 points in the ON state and −0.4 to 2.2 points in the OFF state—none of which met clinical relevance thresholds. The Disability (UPDRS-II) score showed no improvement in the ON state and variable, marginal improvements (0.3–1.8 points) in the OFF state. QoL outcomes were assessed in only one trial (Hauser et al., 2008), but quantitative results were not reported, limiting interpretation. In summary, while istradefylline may modestly reduce OFF-time, its effects on motor function, disability, and QoL are limited and vary between studies. The increased risk of dyskinesia in some patients highlights the need for careful individualization and further long-term safety studies.

#### 3.1.3 Low-quality or insufficient evidence

##### 3.1.3.1 Levodopa–carbidopa controlled release

High-quality data are lacking to support the use of levodopa–carbidopa controlled-release (CR) formulations to treat motor fluctuations in people with PD receiving (attempted) optimal oral levodopa therapy. Three RCTs met the inclusion criteria for this intervention (Hutton et al., 1988; Wolters et al., 1992; Wolters and Tesselaar, 1996)—all evaluated CR formulations against immediate-release (IR) levodopa–carbidopa for the reduction of OFF-time. One study found no difference between CR and placebo (Hutton et al., 1988), whereas the remaining two trials reported modest reductions in OFF time with CR formulations (Wolters et al., 1992; Wolters and Tesselaar, 1996). However, these findings were undermined by substantial methodological limitations, including a high risk of bias and imprecise reporting of outcomes.

None of the studies demonstrated a significant improvement in ON-time duration or functional outcomes, and disability scores remained unchanged. Overall, the quality of evidence was low, and the clinical effect—if present—did not meet the thresholds for a minimal clinically important difference. These findings suggest that while CR formulations may offer minor pharmacokinetic advantages, they do not translate into meaningful therapeutic benefits for motor fluctuations in routine clinical practice.

##### 3.1.3.2 Selegiline

Evidence remains too limited to establish the efficacy of selegiline in treating motor fluctuations in people with PD on (attempted) optimized oral levodopa therapy. Five trials met the inclusion criteria (Heinonen et al., 1989; Teychenne and Parker, 1989; Hubble et al., 1993; Waters et al., 2004; Ondo et al., 2007). Four placebo-controlled studies evaluated the effect of selegiline on OFF-time duration (Heinonen et al., 1989; Hubble et al., 1993; Waters et al., 2004; Ondo et al., 2007). Of these, only one trial demonstrated a reduction in OFF-time of clinical value, with at least a 1-h improvement (Waters et al., 2004). Another study reported statistically significant benefits in end-of-dose and early-morning akinesia, as well as a prolonged effect of each levodopa dose, though the improvement fell short of clinical relevance (Heinonen et al., 1989). Two trials found no significant benefit in reducing OFF time compared with placebo (Heinonen et al., 1989; Ondo et al., 2007).

Three studies assessed changes in ON-time (Hubble et al., 1993; Waters et al., 2004; Ondo et al., 2007). One trial demonstrated a clinically relevant increase in dyskinesia-free ON-time (Waters et al., 2004), whereas another reported a modest improvement only at week 12, not at earlier time points (Ondo et al., 2007). A third trial found no difference in ON-time duration (Hubble et al., 1993). Motor impairment outcomes were evaluated inconsistently, with mixed results across three trials (Heinonen et al., 1989; Teychenne and Parker, 1989; Hubble et al., 1993). Only one study demonstrated improvement in disability scores using the Columbia University Rating Scale (Heinonen et al., 1989). Another study, which used the UPDRS-II and the Schwab and England Scale, found no difference between selegiline and placebo (Hubble et al., 1993). Overall, the quality of evidence was low, with small sample sizes, methodological variability, and heterogeneous results. These limitations preclude strong conclusions regarding the efficacy of selegiline for managing motor fluctuations in PD.

##### 3.1.3.3 Nicotine

Findings to date are insufficient to draw firm conclusions about the efficacy of nicotine in treating motor fluctuations in people with PD on (attempted) optimized oral levodopa therapy. A single open-label trial evaluated transdermal nicotine therapy compared with no additional treatment (Villafane et al., 2018). The study found no significant differences in MDS-UPDRS-III scores between the off-medication and on-medication states. However, improvements in secondary, unblinded outcomes were observed, including better disability scores (UPDRS-II) and daily functioning, as well as reductions in dyskinesia (UPDRS-IV).

No changes in OFF-time or ON-time duration were assessed. Due to the lack of a placebo control and the potential for expectancy bias, findings are considered exploratory. Larger, double-blind, placebo-controlled studies are needed to evaluate nicotine’s potential therapeutic role.

##### 3.1.3.4 Terguride

Existing studies provide inconclusive evidence for the efficacy of terguride in the treatment of motor fluctuations in people with PD receiving (attempted) optimised oral levodopa therapy. A single placebo-controlled trial evaluated terguride in patients with motor fluctuations (Pacchetti et al., 1993). The study found no significant improvements in OFF-time duration or motor scores, as measured by the Columbia University Rating Scale, in either the ON or OFF medication states. Disability and ON-time outcomes were not meaningfully affected, and no increase in dyskinesia risk was observed. Given the lack of clinically significant findings and the absence of confirmatory studies, current evidence does not support the use of terguride for the management of motor fluctuations in PD.

##### 3.1.3.5 Perampanel

Available data are inadequate to support the use of perampanel in managing motor fluctuations in people with PD on (attempted) optimised oral levodopa therapy. Two placebo-controlled trials evaluated perampanel for the treatment of motor fluctuations (Eggert et al., 2010; Lees et al., 2012). Neither study demonstrated superiority over placebo in reducing OFF-time. Only one trial assessed ON-time and disability, with no significant improvement reported. Motor symptoms in the off-medication state, functional capacity, and HR-QoL were not assessed in either trial. The limited scope and inconclusive findings preclude a recommendation for perampanel use in this context.

### 3.2 Surgical and experimental treatments for motor fluctuations in Parkinson’s disease

#### 3.2.1 High-quality evidence

##### 3.2.1.1 Internal globus pallidus deep brain stimulation (GPi-DBS)

Bilateral internal globus pallidus (GPi) deep brain stimulation is likely efficacious for treating motor fluctuations in people with PD on (attempted) optimised oral levodopa therapy. Five studies met eligibility criteria, including two large trials with extended follow-up (Anderson et al., 2005; Weaver et al., 2009; Follett et al., 2010; Williams et al., 2010; Odekerken et al., 2013) and two additional reports from the same cohorts at 36 months (Weaver et al., 2012; Sidiropoulos et al., 2016). The Veterans Affairs CSP 468 study compared DBS (61 GPi, 60 subthalamic nucleus [STN]) with the best medical therapy (BMT) in 255 participants (Weaver et al., 2009). While the surgical target was masked to participants, awareness of receiving surgery versus BMT introduced a risk of bias. Nevertheless, DBS resulted in substantial improvements over BMT: a 4.6-h increase in ON-time without troublesome dyskinesia, a 10.6-point improvement in UPDRS-III (off-medication), a 4.6-point improvement in UPDRS-II, and a 12-point improvement in PDQ-39 scores.

A second phase of the same study directly compared bilateral GPi-DBS (n = 152) and STN-DBS (n = 147) (Follett et al., 2010), finding comparable motor improvements at 24 months. GPi-DBS improved UPDRS-III (off-medication, on-stimulation) by 11.8 points (95% CI 9.5–14.1), while STN-DBS showed a 10.7-point gain (95% CI 8.5–12.9). GPi-DBS was associated with a trend toward greater improvement in ON-time and HR-QoL. At 36 months, both targets maintained benefits, although the STN group showed a more rapid decline in MDRS scores, possibly due to worse baseline cognitive function (Weaver et al., 2012). Two other double-blind trials directly compared GPi versus STN DBS. One study (n = 128) reported no significant difference in functional disability at 12 months, although the STN group showed numerically larger improvements in UPDRS-II, UPDRS-III, and PDQ-39 scores (Odekerken et al., 2013). Specifically, the off-medication UPDRS-III improvement was 20.3 points with STN-DBS versus 11.4 with GPi-DBS (P = 0.03). At 36 months, this gap persisted (Odekerken et al., 2013), but no differences were observed in cognition, mood, or behaviour.

A smaller pilot trial (n = 20) found that both GPi and STN stimulation improved off-medication UPDRS-II and -III scores (Anderson et al., 2005). Taken together, the evidence supports the likely efficacy of GPi-DBS in reducing motor fluctuations, with results similar to those of STN-DBS but slightly less robust. Its favourable cognitive profile may support its use in patients at risk of neurocognitive decline.

#### 3.2.2 Moderate-quality evidence

##### 3.2.2.1 Pallidotomy

Unilateral pallidotomy is likely efficacious for improving off-medication motor impairment in people with PD compared with the best medical therapy. However, evidence remains scarce and inconclusive to support its use over STN-DBS or subthalamotomy. Four trials evaluated unilateral pallidotomy for the management of motor fluctuations (De Bie et al., 1999; Vitek et al., 2003; Esselink et al., 2004; Esselink et al., 2006; Coban et al., 2009). Two randomised trials compared pallidotomy to medical therapy and found statistically and clinically significant improvements in off-medication UPDRS-III scores (approximately a 15-point difference at 6 months), with no improvement in the control groups (De Bie et al., 1999; Vitek et al., 2003). Disability outcomes (UPDRS-II and Schwab-England Scale) also improved significantly in the pallidotomy groups. However, no trials included a sham control, and sample sizes were small.

Only one of these studies assessed OFF-time and ON-time, limiting conclusions on fluctuation metrics (De Bie et al., 1999). In contrast, a trial comparing unilateral pallidotomy with bilateral STN-DBS reported greater improvements with STN-DBS at both 6 months (UPDRS-III: 19 vs. 7 points) and 12 months (24 vs. 12 points) (Esselink et al., 2004; Esselink et al., 2006), favouring DBS as the more effective approach.

A small trial (n = 10) compared unilateral pallidotomy with unilateral subthalamotomy and found similar improvements in off-medication motor scores and daily living function, though the study lacked power to draw definitive conclusions (Coban et al., 2009). Despite encouraging results from early studies, the role of pallidotomy remains limited by the lack of contemporary sham-controlled trials, small sample sizes, and variability in outcome assessment. While the approach may offer benefits in select cases where DBS is contraindicated, current data support its use with caution.

#### 3.2.3 Low-quality or insufficient evidence

##### 3.2.3.1 Subthalamotomy

The evidence base remains inadequate to confirm the efficacy of unilateral or bilateral subthalamotomy for treating motor fluctuations in people with PD. Reported rates of severe hemiballismus—ranging from 20% to 50%—raise significant safety concerns. Two small trials met the eligibility criteria (Merello et al., 2008; Coban et al., 2009). One trial (n = 10) compared unilateral subthalamotomy with unilateral pallidotomy and found that both procedures improved off-medication motor impairment and activities of daily living, with no significant differences between groups (Coban et al., 2009). A second trial (n = 16) compared bilateral subthalamotomy, unilateral subthalamotomy plus contralateral STN-DBS, and bilateral STN-DBS (Merello et al., 2008). All interventions improved off-medication motor scores and functional scales; however, no significant differences were observed between groups.

Critically, both trials reported high rates of hemiballismus—20% in unilateral procedures and up to 50% in bilateral cases—highlighting a major safety concern. No studies compared subthalamotomy to best medical therapy (BMT), and long-term efficacy and cognitive outcomes remain unexplored. Given the small sample sizes, the absence of control arms, and significant adverse effects, the current evidence does not support the routine clinical use of subthalamotomy for the management of motor fluctuations in patients with PD.

##### 3.2.3.2 Zona incerta deep brain stimulation (ZI-DBS)

The strength of evidence is insufficient to recommend the use of bilateral zona incerta deep brain stimulation for the treatment of motor fluctuations in people with PD on (attempted) optimized oral levodopa therapy. One small trial (n = 19) compared bilateral ZI-DBS with the best medical therapy in individuals with Parkinson’s disease (Blomstedt et al., 2018). At 6 months, participants in the ZI-DBS group showed a 41% improvement in off-medication UPDRS-III scores, particularly in tremor control, whereas no change was observed in the BMT group. The quality of life improved from baseline in both groups, but there were no statistically significant differences between them.

Due to the unblinded nature of the intervention—patients were aware of treatment allocation—the study was at high risk of bias despite blinded outcome assessment for motor scores. Given the small sample size, short follow-up, and lack of replication, ZI-DBS cannot be recommended based on current evidence.

##### 3.2.3.3 Glial cell-derived neurotrophic factor (GDNF)

There is currently insufficient evidence to support the use of glial cell-derived neurotrophic factor (GDNF) for treating motor fluctuations in people with PD receiving (attempted) optimised oral levodopa therapy. One randomised trial compared bilateral intraputaminal infusions of GDNF (120 μg per putamen) with placebo in patients with moderate PD (Whone A. et al., 2019). After 40 weeks, there was no significant difference in off-medication UPDRS-III scores between groups. An open-label extension also failed to demonstrate clinical improvement at 80 weeks (Whone A. L. et al., 2019).

Disability outcomes (UPDRS-II) and quality of life (PDQ-39 and EQ-5D) did not improve with GDNF compared with placebo. No differences were observed in dyskinesia severity (UPDRS-IV), and diary-based measures of OFF-time, good-quality ON-time, and troublesome dyskinesia also remained unchanged in both phases of the trial. Although GDNF holds biological plausibility as a neurorestorative therapy, clinical trials have yet to show a meaningful therapeutic effect. Larger, longer-term studies with optimised delivery methods may be necessary to clarify its potential.

## 4 Unequal access to motor fluctuation therapies: insights from Latin American markets

Marked differences were observed in the availability and affordability of pharmacological treatments for motor fluctuations in Parkinson’s disease across Latin American countries when compared with the United States. Overall, the United States had the widest therapeutic portfolio, with 19 drugs authorised for marketing, whereas the number in Latin American countries ranged from 13 in Argentina to only 4 in Peru. Many advanced therapies—such as IPX066, opicapone, LCIG, foslevodopa, foscarbidopa, and istradefylline—were available only in the United States, highlighting a significant access gap between high- and middle-income regions. Other drugs, including safinamide, zonisamide, and ropinirole, were found in only one of the selected Latin American countries, further illustrating the uneven distribution of therapeutic options across the region. This limited regulatory availability constrains treatment choices and may delay the adoption of newer or adjunctive agents with demonstrated efficacy in reducing motor fluctuations.

Price comparisons based on the cost per DDD revealed wide disparities both between and within countries (Table 3). Although some drugs, such as selegiline, rotigotine, and perampanel, were generally less expensive in Latin America than in the United States, others—particularly advanced dopaminergic therapies—showed disproportionately high prices relative to purchasing-power parity. For instance, entacapone and controlled-release levodopa–carbidopa were substantially more costly in most Latin American markets than in the United States after PPP adjustment, indicating that such formulations remain largely unaffordable for many patients. Conversely, some countries, such as Chile and Brazil, exhibited comparatively lower PPP-adjusted prices across several molecules, suggesting stronger public pricing control mechanisms or broader generic competition. Notably, pramipexole ER was generally more affordable in Latin America than in the United States, although the price per DDD remained high; interestingly, in Colombia, it was the only available presentation of pramipexole, underscoring a particular market limitation despite the relatively lower cost.

**TABLE 3:** Price per defined daily dose of pharmacological treatments for motor fluctuations in Parkinson’s disease in selected Latin American countries.

Drug	ATC code	DDD (mg)	Price per DDD (U.S. dollars)									
			USA	ARG	BRA	MEX	CHL	COL	GTM	CRI	PER	PAN^§^
Rasagiline	N04BD02	1	3.49 ± 1.86	0.76 ± 0.13	0.96 ± 0.36	3.52 ± 1.04	0.71 ± 0.23	0.92 ± 0.52	2.10	2.60	2.16	2.44
				2.12 ± 0.37	2.15 ± 0.81	6.66 ± 1.97	1.46 ± 0.48	2.51 ± 1.42	4.87	4.13	4.36	5.30
Pramipexole	N04BC05	2.5	2.27 ± 2.29	4.87 ± 2.44	2.03 ± 0.74	5.71 ± 5.55	5.63 ± 2.88		8.92 ± 5.11	8.73 ± 3.47	6.48 ± 1.72	9.32 ± 5.31
				13.64 ± 6.83	4.54 ± 1.66	10.80 ± 10.50	11.60 ± 5.93		20.64 ± 11.82	13.86 ± 5.51	13.11 ± 3.49	20.26 ± 11.55
Pramipexole ER*	N04BC05	2.5	12.47 ± 10.72	7.42 ± 1.55	3.23 ± 0.30	22.29 ± 17.32	4.27 ± 1.60	2.06 ± 0.40	5.75 ± 3.20	11.50 ± 9.14		10.71
				20.78 ± 4.33	7.23 ± 0.66	42.16 ± 32.75	8.80 ± 3.29	5.66 ± 1.09	13.30 ± 7.42	18.25 ± 14.52		23.29
Amantadine	N04BB01	200	1.31	2.99 ± 0.14	0.43	1.73 ± 0.29	0.99	4.30	1.69			
				8.36 ± 0.38	0.96	3.27 ± 0.55	2.05	11.81	3.90			
Rotigotine	N04BC09	6	43.68 ± 28.18	19.52 ± 9.79	4.01 ± 0.32	11.53 ± 7.95	4.69 ± 1.20	3.63 ± 0.01		NF		
				54.65 ± 27.43	8.97 ± 0.71	21.80 ± 15.03	9.66 ± 2.47	9.95 ± 0.02		NF		
LC–CR**	N04BA02	600	3.48 ± 1.09	1.42 ± 0.18	3.02		3.36		6.42 ± 2.12	4.31		
				3.98 ± 0.50	6.75		6.93		14.87 ± 4.90	6.84		
Selegiline	N04BD01	5	1.75	0.27	0.53	3.44	NF		NF		0.83 ± 0.51	
				0.75	1.18	6.51	NF		NF		1.68 ± 1.04	
Perampanel	N03AX22	8	48.27 ± 22.03	11.17 ± 7.86	6.46 ± 1.49	8.88	13.39 ± 4.91				NF	
				31.27 ± 22.01	14.46 ± 3.33	16.79	27.59 ± 10.11				NF	
Apomorphine	N04BC07	20	612.27	193.52 ± 133.15		24.86		9.88				
				541.87 ± 372.82		47.01		27.12				
Entacapone	N04BX02	1000	3.97	12.41 ± 0.08	7.23	8.03			NF			
				34.74 ± 0.24	16.16	15.18			NF			
Nicotine	N07BA01	14	2.40 ± 1.00	1.36 ± 0.71	3.38 ± 1.79	1.33						
				3.80 ± 1.98	7.56 ± 3.99	2.51						
Safinamide	N04BD03	75	42.76 ± 20.29		1.86 ± 0.43			NF				
					4.17 ± 0.96			NF				
Zonisamide	N03AX15	200	1.19 ± 0.56	4.14 ± 0.99		NF						
				11.60 ± 2.76		NF						
Ropinirole	N04BC04	6	0.63 ± 0.09	1.12 ± 0.25		NF	NF					
				3.14 ± 0.69		NF	NF					
IPX066^#^	N04BA02	1140	33.34 ± 12.55									
								
Opicapone	N04BX04	50	35.47 ± 15.70									
								
Foslevodopa-foscarbidopa^†^	N04BA02	780	106.44									
								
Istradefylline^##^	N04CX01	20	50.91 ± 24.00									
								
LCIG**	N04BA02	600	527.40									
								

Prices are expressed in nominal U.S. dollars (up) and as PPP-adjusted values (down, except for the United States, where PPP = 1), based on data available as of June 2025. Values are presented as mean ± SD (SD shown only when more than one drug presentation was available). ATC: Anatomical Therapeutic Chemical classification code. DDD: Defined Daily Dose. LC–CR: Levodopa–carbidopa controlled release. LCIG: Levodopa–carbidopa intestinal gel. *The WHO does not differentiate extended-release (ER) from immediate-release (IR) formulations; therefore, a 1:1 conversion factor was applied. **The DDD for this formulation was considered equivalent to that of IR, levodopa–carbidopa (conversion factor = 1). ^#^DDD, value obtained from Tetrud et al. (2017). ^†^A DDD, conversion factor of 1.3 was applied. ^§^Incomplete information (see Methods). ^##^DDD is not available. NF indicates that the drug was not found in pharmacies despite having marketing authorization. Terguride was excluded because it is not marketed in the United States (available only in Japan at ¥118 per 0.5 mg tablet, approximately $0.8 USD).

Beyond descriptive price comparisons, several economic confounders likely influence these disparities. Market segmentation between public and private sectors, differences in procurement mechanisms, and the timing of generic entry substantially shape regional price structures. As underscored in the WHO guideline on country pharmaceutical pricing policies (WHO, 2020), uncoordinated or fragmented pricing approaches may lead to inequitable outcomes, where public-sector prices are negotiated effectively but private markets remain unregulated or subject to monopolistic practices. Moreover, the limited use of value-based pricing and differential mark-ups across distribution chains further contributes to intra-country variability.

**FIGURE 3:**
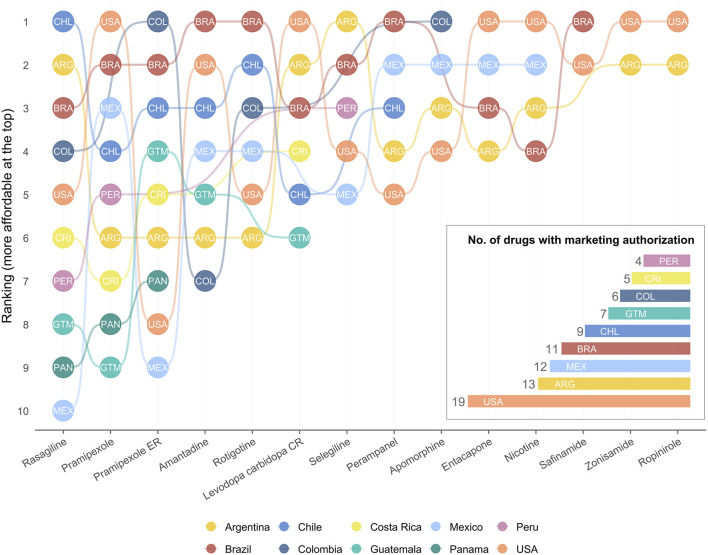
Relative ranking of PPP-adjusted prices per Defined Daily Dose (DDD) of pharmacological treatments for motor fluctuations in Parkinson’s disease across Latin American countries. Each line represents a country, with its position indicating the relative affordability of each drug (1 = lowest PPP-adjusted price per DDD in U.S. dollars). Ranks were calculated within each drug based on the Purchasing Power Parity (PPP)–adjusted price per DDD, allowing cross-country comparisons independent of currency value or income level. Only drugs with available price data in at least one Latin American country are included. Country codes (ISO 3166-1) correspond to those shown in the legend. The nested bar plot on the right shows the number of pharmacological treatments with marketing authorisation in each country, highlighting regional disparities in drug availability. Data for the United States are presented as a reference for international comparison.

Ranking analyses also highlighted heterogeneous patterns (Figure 3). Argentina and Brazil frequently ranked among the most affordable countries for several drugs, whereas smaller markets such as Panama and Guatemala tended to appear near the bottom of the affordability scale. Importantly, no single country combined both wide availability and low cost: Argentina and Mexico, despite their larger number of authorised drugs, displayed substantial price variability, while nations with cheaper options often lacked access to newer agents. These findings underscore the fragmented landscape of antiparkinsonian pharmacotherapy in Latin America, where access is constrained not only by cost but also by regulatory and distributional barriers.

Compared with the United States, Latin American countries as a whole exhibit a pronounced treatment gap. Patients in the region have access to fewer pharmacological alternatives, often at higher relative prices, and frequently rely on older dopaminergic agents. This scenario likely exacerbates inequalities in the management of motor fluctuations, with potential consequences for quality of life and functional independence. According to the WHO report *Improving access to medicines for neurological disorders* (WHO, 2024), these inequities mirror broader systemic weaknesses, including underfunded essential medicines programs, insufficient inclusion of neurological drugs in national lists, and limited capacity for pharmacoeconomic evaluation. Such structural gaps perpetuate the underutilization of effective therapies despite the growing disease burden. Coordinated regional strategies to harmonize drug registration processes, promote the inclusion of newer therapies in national formularies, and ensure equitable pricing—particularly for formulations with proven efficacy in reducing OFF-time—are urgently needed to improve accessibility and continuity of care for patients with Parkinson’s disease in Latin America.

The disparities identified in this analysis are consistent with broader structural barriers described for high-cost and innovative drugs in Latin America. Fragmented health systems, limited universal coverage, and heterogeneous regulatory frameworks continue to hinder equitable access to advanced therapies across the region (Rosselli, 2023). Although initiatives such as centralized procurement and international reference pricing in countries like Brazil, Colombia, and Mexico have achieved partial success in reducing drug prices, these mechanisms remain unevenly implemented and rarely extend to neurological or chronic degenerative disorders such as Parkinson’s disease (Rosselli, 2023). Moreover, while health technology assessment (HTA) agencies—such as CONITEC in Brazil, IETS in Colombia, and CENETEC in Mexico—have strengthened evidence-based decision-making, their influence on pricing and reimbursement decisions for neurological drugs remains limited due to institutional fragmentation and scarce integration across public and private systems (Bas, 2023).

Biosimilars and generics represent a significant yet underutilised opportunity to enhance affordability and reduce reliance on expensive branded agents. However, as noted by Schaffel et al. (2025), the uptake of biosimilars in Latin America has been slow due to heterogeneous approval pathways, limited harmonisation with WHO standards, and weak pharmacovigilance systems. Barriers also include physician reluctance, limited patient awareness, and the absence of clear interchangeability guidelines, which collectively delay market penetration and price competition (Bas, 2023; Schaffel et al., 2025). Coordinated regional strategies—such as joint procurement, simplified HTA procedures, and harmonised regulatory pathways—could enhance cost containment and ensure that the resulting savings are reinvested to expand therapeutic access (Rosselli, 2023; Schaffel et al., 2025).

In addition, the limited adoption of value-based pricing mechanisms further constrains rational price setting in the region. Most Latin American countries still rely on traditional international reference pricing or volume-based discounts, with little linkage between price and therapeutic value. As emphasised by Mejía et al. (2018), the absence of formal cost-effectiveness thresholds, fragmented data systems, and lack of real-world evidence limit the application of value-based reimbursement. Expanding regional collaboration through existing initiatives such as RedETSA or Mercosur could provide a shared framework for evaluating clinical benefit and negotiating fair prices based on value (Mejía et al., 2018; Rosselli, 2023).

In accordance with PAHO’s *Access to High-Cost Medicines in the Americas* report (PAHO, 2023), improving regional coordination through mechanisms such as the Strategic Fund and other pooled procurement platforms could mitigate these barriers by aggregating demand, strengthening negotiating power, and stabilising supply. This approach has proven cost-effective for vaccines and essential medicines and could be expanded to selected neurological treatments. Additionally, regulatory harmonisation across Latin American countries could accelerate market entry for generics and biosimilars while minimising redundant national evaluations. Streamlining approval timelines and adopting mutual recognition procedures would reduce costs for manufacturers and regulators alike, facilitating earlier access to affordable therapies.

From a policy perspective, evidence-based interventions should therefore prioritize regional pooled procurement for high-cost neurological drugs through mechanisms modeled on the PAHO Strategic Fund; harmonization of regulatory and pharmacovigilance frameworks to promote timely generic entry; implementation of transparent value-based pricing policies to link reimbursement to clinical benefit; and integration of neurological disorders into essential medicine benefit packages and national financing schemes, ensuring sustainable inclusion of Parkinson’s therapies in public formularies (WHO, 2020; PAHO, 2023). Such policies would directly address the confounders identified in this study—namely, fragmented markets, delayed generic competition, and lack of coordinated negotiation—transforming descriptive accessibility analyses into actionable regional strategies for equitable therapeutic access.

Finally, the absence of dedicated funding mechanisms for neurological diseases—unlike the special programs for cancer or rare disorders established in Chile (*Ley Ricarte Soto*) and Uruguay (*Fondo Nacional de Recursos*)—further limits patient access to advanced Parkinson’s treatments (Rosselli, 2023). These systemic shortcomings underscore that improving access to antiparkinsonian therapies in Latin America will require not only price negotiations but also regional harmonisation of regulatory and reimbursement frameworks, broader acceptance of biosimilars and generics, and sustained investment in centralised procurement and value-based pricing mechanisms to achieve equitable and sustainable access to care. Incorporating the WHO’s call for intersectoral collaboration and pooled regional procurement (WHO, 2020; WHO, 2024) could promote price transparency, stimulate market competition, and strengthen the sustainability of public drug programs. These actions, grounded in global policy recommendations, would ensure that access to antiparkinsonian therapies aligns with equity principles enshrined in universal health coverage frameworks.

## 5 Perspectives

This review highlights the persistent discrepancy between the therapeutic efficacy reported in clinical trials and the real-world value of treatments for motor fluctuations in Parkinson’s disease, particularly when viewed through the lens of accessibility and functional relevance. While numerous interventions show statistically significant improvements in OFF-time, only a minority translate into consistent functional benefits or meaningful gains in quality of life. Building on a comparative framework, the present synthesis emphasises two complementary therapeutic paradigms—dopaminergic strategies that optimise synaptic dopamine signalling through replacement or prolongation mechanisms—and non-dopaminergic or multimodal strategies that target glutamatergic, adenosinergic, or neurotrophic pathways. This conceptual distinction clarifies trends in efficacy and underscores that pharmacological innovation has plateaued mainly in dopaminergic modulation, while mechanistic diversification remains limited. Ten central challenges emerge from the evidence.

First, across dopaminergic strategies, efficacy in reducing OFF-time does not consistently translate into functional improvement or patient-reported benefit. Although high-efficacy pharmacological agents such as IPX066 (Hauser et al., 2013), opicapone (Ferreira et al., 2016; Lees et al., 2017; Takeda et al., 2021) and safinamide (Borgohain et al., 2014; Schapira et al., 2017; Hattori et al., 2020b; Wei et al., 2022) demonstrate reductions in OFF-time, they fail to yield consistent or clinically important improvements in disability. IPX066, for instance, shows modest functional gains below the threshold of clinical significance and no measurable benefit on HR-QoL (Hauser et al., 2013). Opicapone demonstrates minimal functional benefit despite robust motor effects (Ferreira et al., 2016; Lees et al., 2017; Takeda et al., 2021). Safinamide, approved on the basis of symptomatic control, has never been evaluated using validated functional outcomes, highlighting a troubling absence of patient-centred endpoints in pivotal trials (Borgohain et al., 2014; Schapira et al., 2017; Hattori et al., 2020b; Wei et al., 2022). While several dopaminergic agents are “efficacious” by GRADE criteria, their clinical relevance remains moderate, reflecting a gap between statistical and patient-perceived benefit (de Bie et al., 2025).

Second, when considered across pharmacological classes, improvements in motor scales rarely translate into sustained gains in wellbeing or autonomy. Among high-efficacy agents, only pramipexole yields functional improvements that approach clinical relevance; even then, quality-of-life data findings are variable across studies (Mizuno et al., 2003; Wong et al., 2003; Moller et al., 2005; Poewe et al., 2007; Schapira et al., 2011; Mizuno et al., 2012). Rotigotine (LeWitt et al., 2007; Poewe et al., 2007; Mizuno et al., 2014; Nicholas et al., 2014; Nomoto et al., 2014; Zhang et al., 2017) and safinamide (Borgohain et al., 2014; Schapira et al., 2017; Hattori et al., 2020b; Wei et al., 2022) exhibit either limited or equivocal effects, and opicapone fails to improve HR-QoL altogether. This pattern reflects a broader methodological limitation of PD trials—most were powered for OFF-time or UPDRS outcomes rather than QoL or daily function, leading to efficacy inflation detached from real-world relevance. When benefits in UPDRS-II or OFF-time do not translate into patient-reported gains, the clinical justification for widespread use becomes questionable, particularly in settings with constrained resources.

Third, disparities in access and affordability add a second layer of heterogeneity that shapes therapeutic outcomes beyond pharmacodynamics. The availability of effective treatments in low- and middle-income countries remains unacceptably limited. IPX066 (Hauser et al., 2013), intestinal gel therapy (Olanow et al., 2014; Chung et al., 2022), foslevodopa–foscarbidopa (Soileau et al., 2022), and apomorphine infusion (Hattori et al., 2014; Katzenschlager et al., 2018; Olanow et al., 2020) are rarely or never available outside high-income countries. Even mid-tier options like rotigotine (LeWitt et al., 2007; Poewe et al., 2007; Mizuno et al., 2014; Nicholas et al., 2014; Nomoto et al., 2014; Zhang et al., 2017) or safinamide (Borgohain et al., 2014; Schapira et al., 2017; Hattori et al., 2020b; Wei et al., 2022) remain out of reach for most LMICs, and their inclusion in treatment guidelines risks reinforcing inequity. While pramipexole (Mizuno et al., 2003; Wong et al., 2003; Moller et al., 2005; Poewe et al., 2007; Schapira et al., 2011; Mizuno et al., 2012) and rasagiline (Parkinson Study Group, 2005; Rascol et al., 2005; Zhang L. et al., 2013; Hattori et al., 2018; Zhang et al., 2018) are more accessible, they are still inconsistently distributed through public systems. As shown in the Latin American market analysis of this review, such inequities are compounded by fragmented procurement and differential timing of generic entry, which limit price competition and perpetuate therapeutic gaps despite guideline endorsement. This access gap undermines the therapeutic value of evidence-based recommendations and perpetuates structural disparities in Parkinson’s disease care.

Fourth, functional and QoL outcomes remain inconsistently reported or underpowered, limiting the interpretability of apparent motor benefits, even in trials supporting regulatory approval. Of all the pharmacological interventions reviewed, only a fraction report both OFF-time and functional or HR-QoL outcomes. Entacapone (Parkinson Study Group, 1997; Rinne et al., 1998; Poewe et al., 2002; Brooks et al., 2003; Fenelon et al., 2003; Reichmann et al., 2005; Deuschl et al., 2007; Lew et al., 2011), zonisamide (Murata et al., 2007; Murata et al., 2015), and amantadine ER (Ory-Magne et al., 2014; Oertel et al., 2017; Pahwa et al., 2017) exemplify this issue: their OFF-time benefits are modest, and neither functional scores nor patient-reported outcomes are systematically evaluated. This undermines clinical decision-making, particularly for patients and clinicians who prioritise outcomes reflecting real-world functioning.

Fifth, heterogeneity in trial design and sponsorship introduces systematic bias across the evidence base. Small sample sizes, the lack of sham or active controls, and heterogeneous methodologies often compromise the quality of the evidence supporting moderate- and low-tier treatments. The trials varied in patient inclusion criteria (early vs. advanced fluctuations), comparator types (placebo vs. best medical therapy), follow-up duration, and the definition of primary endpoints (OFF-time, ON-time, or UPDRS-based disability). Publication bias likely skews the literature toward positive findings, particularly in industry-sponsored trials. Such biases may partly explain why nearly all dopaminergic adjuncts reached at least a “likely efficacious” rating despite modest between-drug differences. The evidence base for commonly used agents such as ropinirole (Rascol et al., 1996; Lieberman et al., 1998; Brunt et al., 2002; Im et al., 2003; Barone et al., 2007; Mizuno et al., 2007; Pahwa et al., 2007; Watts et al., 2010; Reichmann et al., 2011; Stocchi et al., 2011; Zhang Z. et al., 2013; Mizuno et al., 2014; Zesiewicz et al., 2017; Hattori et al., 2020a) or entacapone (Parkinson Study Group, 1997; Rinne et al., 1998; Poewe et al., 2002; Brooks et al., 2003; Fenelon et al., 2003; Reichmann et al., 2005; Deuschl et al., 2007; Lew et al., 2011) is derived from heterogeneous and often contradictory trials. Similarly, selegiline (Heinonen et al., 1989; Teychenne and Parker, 1989; Hubble et al., 1993; Waters et al., 2004; Ondo et al., 2007) and levodopa–carbidopa controlled release (Hutton et al., 1988; Wolters et al., 1992; Wolters and Tesselaar, 1996) continue to be prescribed despite consistently weak evidence and no meaningful benefit on OFF-time, function, or HR-QoL. Terguride (Pacchetti et al., 1993) and perampanel (Eggert et al., 2010; Lees et al., 2012) offer no demonstrable efficacy but are still occasionally cited as adjuncts in refractory cases, reflecting the inertia of outdated clinical practice.

Sixth, device-aided and surgical interventions illustrate the upper limit of dopaminergic substitution yet expose deep socioeconomic divides. Surgical therapies, while supported by higher-quality evidence in the case of GPi-DBS (Anderson et al., 2005; Weaver et al., 2009; Follett et al., 2010; Williams et al., 2010; Weaver et al., 2012; Odekerken et al., 2013; Sidiropoulos et al., 2016), remain inaccessible for the vast majority of patients worldwide. GPi-DBS improves both motor and functional outcomes, with measurable gains in PDQ-39 scores, but is restricted mainly to tertiary centres in high-income countries. Pallidotomy (de Bie et al., 1999; Vitek et al., 2003; Esselink et al., 2004; Esselink et al., 2006; Coban et al., 2009) offers a lower-cost alternative with moderate efficacy and safer profiles than subthalamotomy (Merello et al., 2008; Coban et al., 2009), yet remains underutilized, despite being technically feasible in LMICs. Subthalamotomy (Merello et al., 2008; Coban et al., 2009) and zona incerta DBS (Blomstedt et al., 2018), by contrast, suffer from poor safety profiles and a high risk of bias, making them unsuitable substitutes.

Seventh, experimental neurorestorative strategies have so far failed to transcend proof-of-concept. GDNF therapy, despite biological plausibility, has not demonstrated clinically relevant benefit (Whone A. et al., 2019; Whone A. L. et al., 2019). The absence of clinical effects and lack of improvement in patient outcomes—despite demonstrated safety—serves as a cautionary tale about over-reliance on neuroprotective mechanisms without clear symptomatic impact. Such strategies may redirect resources without offering tangible benefits to patients, particularly when basic symptomatic therapies remain out of reach in large parts of the world.

Eighth, translating global evidence into local policy remains hindered by a disconnect between efficacy and feasibility. Regulatory approval and treatment guidelines often fail to incorporate access as a critical determinant of clinical utility. The present Latin American analysis underscores how therapies recommended in international guidelines—such as IPX066 (Hauser et al., 2013) or LCIG (Olanow et al., 2014; Chung et al., 2022)—may be clinically sound yet practically unattainable across most middle-income contexts. As emphasised in this review, real-world therapeutic impact depends on national regulatory capacity, procurement efficiency, and pricing transparency rather than on efficacy alone—an issue absent from most evidence hierarchies. Without integrating cost and availability considerations into policy and guideline development, evidence-based medicine risks becoming aspirational rather than actionable.

Ninth, comparative synthesis across pharmacological classes reveals redundancy rather than complementarity. Few trials include head-to-head comparisons between agents, limiting the clinician’s ability to choose based on comparative effectiveness or tolerability. Aggregated evidence suggests that differences among dopamine agonists or among COMT and MAO-B inhibitors are minor and likely overshadowed by patient-specific tolerability and adherence factors. Therapeutic sequencing is frequently driven by availability or historical preference rather than data. Given the redundancy in mechanisms across many dopaminergic agents, comparative data are essential to optimize therapy, reduce polypharmacy, and guide rational treatment planning (Ortega-Robles et al., 2025).

Tenth, structural inequities continue to mediate access, reinforcing the divide between innovation and implementation. There is insufficient investment in implementation science, health systems strengthening, and locally relevant research to close the access gap in LMICs. The scarcity of foundational treatments in these settings is not merely a pharmaceutical supply issue—it reflects broader deficiencies in financing, infrastructure, and policy. The Latin American experience exemplifies this gap: fragmented procurement systems, limited regional cooperation, and weak HTA integration collectively hinder equitable access. Beyond price, evidence synthesis rarely accounts for health-system readiness, a limitation echoed here in the context of Latin America’s uneven regulatory and distributional capacity (de Bie et al., 2025). Furthermore, the predominance of trials conducted in high-income settings limits the generalizability of efficacy estimates to regions with constrained drug availability and health system infrastructure. Unless these structural barriers are addressed through coordinated regional frameworks and sustainable pricing mechanisms, even the most compelling clinical evidence will fail to translate into meaningful patient benefit.

In summary, while a broad range of treatments is available for motor fluctuations in PD, the clinical utility of many interventions remains constrained by modest functional benefits, minimal impact on QoL, and pervasive inequities in access. Reframing evidence through the dopaminergic–non-dopaminergic model clarifies that advances have optimised mainly the delivery of the same neurotransmitter system rather than diversifying therapeutic targets. The current model of innovation without implementation widens the gap between what is possible and what is achievable—particularly for patients in low- and middle-income settings. Bridging this divide will require integrating accessibility analyses into future evidence syntheses, promoting context-adapted guidelines, and developing regionally coordinated strategies that link therapeutic value to affordability and feasibility, ensuring that clinical progress in PD becomes globally inclusive.
