# Supplementary material for: Treatment strategies for motor fluctuations in Parkinson’s disease: a systematic review of efficacy, functionality, and drug accessibility with a focus on Latin America
Source: Front Pharmacol. 2025 Dec 3;16:1725248. doi: 10.3389/fphar.2025.1725248 (PMC12708893; doi:10.3389/fphar.2025.1725248)
Supplement: Supplementary file 1 [file Supplementaryfile1.docx]

Supplementary Material

# Search strategy

PubMed (via MEDLINE) and Scopus were searched from 1990/01/01 to 2025/05/25. No language or publication-type limits were imposed at query time to maximise sensitivity; design and population filters were applied during screening. Reference lists of eligible studies and recent reviews were hand-searched to identify additional articles. This strategy is consistent with PRISMA 2020 and the review scope outlined in the protocol. Three concept blocks were combined: (i) Parkinson's disease, (ii) motor fluctuations (wearing-off/OFF-time/ON-time/dyskinesia), and (iii) interventions of interest (adjunct pharmacotherapies, device-aided continuous dopaminergic delivery, and surgery). Study-design terms were included to surface randomised and controlled comparative evidence while retaining high recall; final inclusion was determined during screening.

**PubMed (MEDLINE):**

("Parkinson Disease"[Mesh] OR parkinson*[tiab]) AND ("Motor Fluctuations"[tiab] OR "wearing off"[tiab] OR wearing-off[tiab] OR "OFF time"[tiab] OR OFF-time[tiab] OR "ON time"[tiab] OR on-time[tiab] OR dyskinesia*[tiab]) AND (levodopa[tiab] OR "carbidopa levodopa"[tiab] OR IPX066[tiab] OR Rytary[tiab] OR opicapone[tiab] OR entacapone[tiab] OR tolcapone[tiab] OR rasagiline[tiab] OR safinamide[tiab] OR selegiline[tiab] OR pramipexole[tiab] OR ropinirole[tiab] OR rotigotine[tiab] OR amantadine[tiab] OR istradefylline[tiab] OR zonisamide[tiab] OR apomorphine[tiab] OR "levodopa carbidopa intestinal gel"[tiab] OR LCIG[tiab] OR foslevodopa[tiab] OR foscarbidopa[tiab] OR "deep brain stimulation"[tiab] OR DBS[tiab] OR pallidotomy[tiab] OR subthalamotomy[tiab]) NOT (animals[mh] NOT humans[mh])

**Scopus:**

TITLE-ABS-KEY( ( (parkinson* W/2 disease) OR PD ) AND ( "motor fluctuat*" OR "motor complication*" OR (wearing W/1 off) OR "wearing-off" OR "end-of-dose" OR (off W/1 time) OR "off-time" OR (on W/1 time) OR "on-time" OR "on/off" OR "on-off" OR ("OFF" W/2 episode*) OR ("OFF" W/2 period*) OR ("OFF" W/2 state*) OR ("levodopa-induced" W/2 dyskinesi*) OR dyskinesi* ) AND ( levodopa OR (carbidopa W/1 levodopa) OR ipx066 OR Rytary OR ipx203 OR "extended-release" W/3 levodopa OR Inbrija OR "levodopa inhalation" OR opicapone OR entacapone OR tolcapone OR rasagiline OR safinamide OR selegiline OR pramipexole OR ropinirole OR rotigotine OR amantadine OR Gocovri OR "Osmolex ER" OR istradefylline OR zonisamide OR apomorphine OR Kynmobi OR Apokyn OR ("levodopa carbidopa intestinal" W/2 (gel OR suspension)) OR LCIG OR Duodopa OR Duopa OR foslevodopa OR foscarbidopa OR ABBV-951 OR ND0612 OR (subcutaneous W/3 levodopa) OR (continuous W/3 levodopa W/3 infusion) OR "deep brain stimulation" OR DBS OR STN OR GPi OR pallidotomy OR subthalamotomy ) ) AND ( TITLE-ABS-KEY(human*) OR TITLE-ABS-KEY(patient*) ) AND NOT TITLE-ABS-KEY(mice OR mouse OR murine OR rat OR rats OR rodent* OR primate* OR macaque* OR canine OR dog OR feline OR cat OR porcine OR pig OR rabbit*) AND (PUBYEAR > 1990 AND PUBYEAR < 2025).

Records identified (16,064) from:

- PubMed (n = 4,477)
- Scopus (n = 11,587)

Records removed *before screening*:

- Duplicate records removed (n = 3,244)

Records screened

(n = 12,820)

Records excluded

(n = 12,394)

Reports sought for retrieval

(n = 426)

Reports not retrieved

(n = 0)

Reports assessed for eligibility

(n = 426)

Reports excluded (n = 334):

- Not PD motor fluctuations (n = 142)
- No OFF/ON, UPDRS-II, PDQ-39, or EQ-5D outcomes (n = 88)
- Ineligible design (n = 64)
- Follow-up below thresholds (n = 40)

Studies included in review

(n = 92)

**Identification of studies via databases and registers**

**Identification**

**Screening**

**Included**

**Supplementary Figure 1.** PRISMA flow diagram, adapted from: Page MJ, McKenzie JE, Bossuyt PM, Boutron I, Hoffmann TC, Mulrow CD, et al. The PRISMA 2020 statement: an updated guideline for reporting systematic reviews. BMJ 2021;372:n71. Licensed under CC BY 4.0 (http://creativecommons.org/licenses/by/4.0/).

# Official Data Sources Consulted

The following official sources were consulted to obtain the information used in the analysis of drug accessibility in Latin America. All webpages were accessed in July 2025:

- **International Monetary Fund (IMF)** – Exchange rates.
  <https://data.imf.org/en/Data-Explorer?datasetUrn=IMF.STA:ER(4.0.1)>
- **World Health Organization Collaborating Centre for Drug Statistics Methodology** – ATC/DDD Index.
  <https://atcddd.fhi.no/atc_ddd_index/>
- **World Bank** – Purchasing Power Parity (PPP) conversion factor indicator.
  <https://data.worldbank.org/indicator/PA.NUS.PPP>

**Supplementary Table 1.** Official Regulatory Agencies and Their Websites for Each Country Included in the Study

| Country | Regulatory agency | Acronym | Consultation website |
| --- | --- | --- | --- |
| Mexico | Comisión Federal para la Protección contra Riesgos Sanitarios | COFEPRIS | <https://tramiteselectronicos02.cofepris.gob.mx/BuscadorPublicoRegistrosSanitarios/BusquedaRegistroSanitario.aspx> |
| Argentina | Administración Nacional de Medicamentos, Alimentos y Tecnología Médica | ANMAT | <https://servicios.pami.org.ar/vademecum/views/consultaPublica/listado.zul> |
| Brazil | Agência Nacional de Vigilância Sanitária | ANVISA | <https://consultas.anvisa.gov.br/#/medicamentos/> |
| Chile | Agencia Nacional de Medicamentos | ANAMED | <https://registrosanitario.ispch.gob.cl/> |
| Colombia | Instituto Nacional de Vigilancia de Medicamentos y Alimentos | INVIMA | <https://consultaregistro.invima.gov.co/Consultas/consultas/consreg_encabcum.jsp> |
| Peru | Dirección General de Medicamentos, Insumos y Drogas | DIGEMID | <https://www.digemid.minsa.gob.pe/rsProductosFarmaceuticos/> |
| Guatemala | Departamento de Regulación y Control de Productos Farmacéuticos y Afines | DRCPFA | <https://medicamentos.mspas.gob.gt/index.php/consultas/registros-vigentes> |
| Costa Rica | Dirección de Regulación de Productos de Interés Sanitario | DRPIS | <https://registrelo.go.cr/reports/62> |
| Panama | Dirección Nacional de Farmacia y Drogas | DNFD | <https://tramites-minsa.panamadigital.gob.pa/sirfad/VALIDADOR/> |
| USA | Food and Drug Administration | FDA | <https://www.accessdata.fda.gov/scripts/cder/daf/> |

# Sources of Drug Price and Regulatory Data

The following sources were consulted to obtain retail prices and official reference data for antiparkinsonian medications included in the Latin American accessibility analysis. When applicable, pharmacies were selected based on national distribution, reliability, and breadth of their online catalogues. Official regulatory or governmental sources were used when available to verify reference or maximum retail prices.

**Mexico:**

- **Farmacias San Pablo, S.A. de C.V.** — [www.farmaciasanpablo.com.mx](https://www.farmaciasanpablo.com.mx)
  One of Mexico's largest retail pharmacy chains, with extensive online and in-store coverage nationwide.
- **Grupo Farmacéutico del Ahorro, S.A. de C.V.** — [fahorro.com](https://www.fahorro.com)
  Major pharmacy network with broad geographic presence and a comprehensive online catalog.
- **Operadora de Farmacias Guadalajara, S.A. de C.V.** — [www.farmaciasguadalajara.com](https://www.farmaciasguadalajara.com)
  National pharmacy chain with both retail and online sales; widely distributed across states.
- **Signufarma S.A. de C.V.** — [signufarma.com.mx](https://signufarma.com.mx)
  Online pharmacy with a wide catalogue and national shipping.
- **Farmacia Coyoacán** — [farmaciacoyoacan.com](https://farmaciacoyoacan.com)
  Mid-sized pharmacy with regional distribution; included for comparison of independent outlets.
- **Pharma Club, S.A. de C.V.** — [www.pharmaclub.com.mx](https://www.pharmaclub.com.mx)
  National online pharmacy offering prescription and over-the-counter medications; included as an additional representative e-commerce source for retail pricing in Mexico.

**Argentina:**

- **Droguería Zeta S.R.L.** — [www.drzeta.com.ar](https://www.drzeta.com.ar)
  Major online pharmacy and drug wholesaler offering prescription medications nationwide.
- **Precios de Medicamentos Argentina** — [preciosdemedicamentos.com.ar](https://preciosdemedicamentos.com.ar)
  Independent price comparison portal aggregating retail pharmacy prices.
- **Alfabeta S.A.C.I.F. y S.** — [www.alfabeta.net](https://www.alfabeta.net)
  The commercial pharmaceutical database is widely used for national price reference.
- **Listado de Precios de Referencia del Ministerio de Salud** — [www.argentina.gob.ar/precios-de-medicamentos](https://www.argentina.gob.ar/precios-de-medicamentos)
  Official government list providing reference prices for prescription medicines.

**Brazil:**

- **Listas de Preços de Medicamentos do Ministério da Saúde (ANVISA/CMED)** — [www.gov.br/anvisa/pt-br/assuntos/medicamentos/cmed/precos](https://www.gov.br/anvisa/pt-br/assuntos/medicamentos/cmed/precos)
  Official national database of regulated maximum retail prices for pharmaceuticals.
- **Drogaria São Paulo** — [www.drogariasaopaulo.com.br](https://www.drogariasaopaulo.com.br)
  One of the country's largest pharmacy networks with broad national coverage.
- **Drogaria Raia–Drogasil** — [www.drogaraia.com.br](https://www.drogaraia.com.br)
  National chain and online pharmacy, part of Brazil's largest retail pharmacy group.
- **Panvel Farmácias** — [www.panvel.com](https://www.panvel.com)
  Leading Southern Brazil pharmacy chain with an extensive online catalogue.

**Chile:**

- **Salcobrand** — [salcobrand.cl](https://salcobrand.cl)
  One of the three largest national pharmacy chains, offering broad market coverage.
- **Farmacias Ahumada (FASA)** — [www.farmaciasahumada.cl](https://www.farmaciasahumada.cl)
  Major national chain with extensive distribution and online price availability.
- **Cruz Verde** — [www.cruzverde.cl](https://www.cruzverde.cl)
  Chile's largest pharmacy chain is included as a benchmark for national retail pricing.

**Colombia:**

- **Farmacias Pasteur** — [www.farmaciaspasteur.com.co](https://www.farmaciaspasteur.com.co)
  Well-established retail pharmacy with national distribution and online pricing.
- **Farmatodo** — [www.farmatodo.com.co](https://www.farmatodo.com.co)
  National pharmacy chain with both physical and online services.
- **Cruz Verde** — [www.cruzverde.com.co](https://www.cruzverde.com.co)
  One of Colombia's largest pharmacy networks is widely representative of market prices.

**Peru:**

- **Farmacia Universal** — [www.farmaciauniversal.com](https://www.farmaciauniversal.com)
  Longstanding national pharmacy chain with online sales.
- **Inkafarma** — [inkafarma.pe](https://www.inkafarma.pe)
  Peru's largest pharmacy network, with wide national coverage and online catalogue.
- **Mifarma** — [www.mifarma.com.pe](https://www.mifarma.com.pe)
  The second-largest pharmacy chain in Peru, frequently used for national price comparisons.

**Guatemala:**

- **Farmacias Cruz Verde Guatemala** — [cruzverde.com.gt](https://cruzverde.com.gt)
  Major national chain with an extensive network of branches and online availability.
- **Farmacias Batres** — [farmaciasbatres.com](https://farmaciasbatres.com)
  Locally recognised chain with several outlets across the country.
- **Farmacias Galeno** — [www.farmaciasgaleno.com.gt](https://www.farmaciasgaleno.com.gt)
  Regional chain providing prescription drug prices through its online platform.

**Costa Rica:**

- **FarmaValue** — [www.farmavalue.com](https://www.farmavalue.com)
  Regional Central American pharmacy network with online services.
- **Farmacia La Bomba** — [www.farmacialabomba.com](https://www.farmacialabomba.com)
  Well-established national pharmacy chain with publicly listed prices.
- **Farmacia Sucre** — [sucreenlinea.com](https://sucreenlinea.com)
  Local chain with online sales and representative retail prices.

**Panama:**

- **Farmacias El Javillo**
  Traditional national pharmacy with in-store sales; prices were obtained through direct consultation as online listings were not publicly available.

**United States:**

- **National Average Drug Acquisition Cost (NADAC)** — [data.medicaid.gov/dataset/f38d0706-1239-442c-a3cc-40ef1b686ac0](https://data.medicaid.gov/dataset/f38d0706-1239-442c-a3cc-40ef1b686ac0)
  Official U.S. government database providing national reference prices for prescription drugs.
- **SingleCare** — [www.singlecare.com](https://www.singlecare.com)
  Commercial platform aggregating pharmacy discount prices across the United States.
- **GoodRx** — [www.goodrx.com](https://www.goodrx.com)
  Widely used retail price comparison website covering major U.S. pharmacies.
- **AbbVie Pharmaceutical Product Catalogue**
  Manufacturer's official price listings used for reference of proprietary formulations.

# Risk of bias tables

**Supplementary Table 2.** Risk of bias assessment of included studies on pharmacological treatments for motor fluctuations in Parkinson's disease.

| Treatment | References | Random sequence generation | Allocation concealment | Blinding participants and personnel | Blinding outcome assessment | Incomplete outcome data | Selective reporting |
| --- | --- | --- | --- | --- | --- | --- | --- |
| IPX066 | Hauser et al., 2013 | + | + | + | + | + | + |
| Opicapone | Ferreira et al., 2016 | + | + | + | + | + | + |
|  | Lees et al., 2017 | + | + | + | + | ? | + |
|  | Takeda et al., 2021 | + | + | + | + | ? | + |
| Pramipexole IR/ER | Mizuno et al., 2003 | + | + | + | + | + | + |
|  | Wong et al., 2003 | + | – | + | + | + | + |
|  | Moller et al., 2005 | + | ? | + | + | + | – |
|  | Poewe et al., 2007 | + | + | + | + | – | + |
|  | Schapira et al., 2011 | + | + | + | + | + | – |
|  | Mizuno et al., 2012 | + | ? | + | + | + | + |
| Rotigotine | LeWitt et al., 2007 | + | + | + | + | + | + |
|  | Poewe et al., 2007 | + | + | + | + | – | + |
|  | Mizuno et al., 2014 | + | ? | + | + | + | + |
|  | Nicholas et al., 2014 | + | + | + | + | ? | ? |
|  | Nomoto et al., 2014 | + | ? | + | + | + | + |
|  | Zhang et al., 2017 | + | + | + | + | + | + |
| Safinamide | Borgohain et al., 2014 | + | + | + | + | + | + |
|  | Schapira et al., 2017 | + | + | + | + | + | ? |
|  | Hattori et al., 2020b | + | ? | + | + | ? | + |
|  | Wei et al., 2022 | + | + | + | + | + | + |
| Levodopa–carbidopa intestinal gel | Olanow et al., 2014 | + | + | + | + | ? | ? |
|  | Chung et al., 2022 | ? | ? | – | – | – | + |
| Foslevodopa–foscarbidopa subcutaneous | Soileau et al., 2022 | + | + | + | + | + | + |
| Apomorphine infusion | Hattori et al., 2014 | ? | ? | ? | + | + | + |
|  | Katzenschlager et al., 2018 | + | + | ? | + | – | + |
|  | Olanow et al., 2020 | + | + | + | + | – | + |
| Rasagiline | Parkinson Study Group, 2005 | + | ? | – | – | – | + |
|  | Rascol et al., 2005 | + | + | + | + | + | + |
|  | Zhang et al., 2013a | + | + | + | + | + | ? |
|  | Hattori et al., 2018 | + | ? | + | + | ? | + |
|  | Zhang et al., 2018 | + | + | + | + | + | + |
| Zonisamide | Murata et al., 2007 | + | + | + | + | – | + |
|  | Murata et al., 2015 | + | + | + | + | + | ? |
| Ropinirole IR | Rascol et al., 1996 | + | ? | ? | + | + | + |
|  | Lieberman et al., 1998 | + | ? | + | + | + | + |
|  | Brunt et al., 2002 | ? | ? | ? | ? | ? | ? |
|  | Im et al., 2003 | ? | ? | ? | ? | ? | + |
|  | Barone et al., 2007 | + | + | + | + | ? | + |
|  | Mizuno et al., 2007 | + | ? | + | + | ? | + |
|  | Pahwa et al., 2007 | + | + | – | ? | – | + |
|  | Watts et al., 2010 | + | + | + | + | + | – |
|  | Reichmann et al., 2011 | + | + | + | + | – | ? |
|  | Stocchi et al., 2011 | + | + | + | + | + | + |
|  | Zhang et al., 2013b | + | ? | + | + | + | + |
|  | Mizuno et al., 2014 | + | ? | + | + | + | + |
|  | Zesiewicz et al., 2017 | + | + | + | + | ? | + |
|  | Hattori et al., 2020a | + | + | + | + | ? | ? |
| Entacapone | Parkinson Study Group, 1997 | + | + | – | + | + | + |
|  | Rinne et al., 1998 | ? | ? | ? | ? | + | + |
|  | Poewe et al., 2002 | + | + | – | – | + | + |
|  | Brooks et al., 2003 | + | + | + | + | – | + |
|  | Fenelon et al., 2003 | ? | ? | – | ? | ? | + |
|  | Reichmann et al., 2005 | ? | ? | ? | – | – | + |
|  | Deuschl et al., 2007 | ? | ? | – | + | + | + |
|  | Lew et al., 2011 | + | ? | – | ? | + | + |
| Amantadine ER | Ory-Magne et al., 2014 | + | + | ? | ? | – | ? |
|  | Oertel et al., 2017 | + | + | + | + | ? | + |
|  | Pahwa et al., 2017 | + | + | + | + | – | + |
| Istradefylline | Hauser et al., 2003 | + | + | + | + | + | – |
|  | Hauser et al., 2008 | ? | ? | + | + | + | + |
|  | LeWitt et al., 2008 | ? | ? | + | + | + | + |
|  | Stacy et al., 2008 | + | ? | + | + | + | + |
|  | Mizuno et al., 2010 | ? | ? | + | + | + | + |
|  | Pourcher et al., 2012 | + | + | ? | + | + | + |
|  | Mizuno et al., 2013 | ? | ? | + | + | + | + |
|  | Li et al., 2015 | + | ? | ? | + | + | + |
| Levodopa–carbidopa CR | Hutton et al., 1988 | – | – | ? | ? | – | ? |
|  | Wolters et al., 1992 | + | – | – | – | – | + |
|  | Wolters and Tesselaar, 1996 | + | – | + | + | + | + |
| Selegiline | Heinonen et al., 1989 | ? | ? | ? | ? | ? | ? |
|  | Teychenne and Parker, 1989 | + | ? | + | + | + | + |
|  | Hubble et al., 1993 | ? | ? | ? | ? | + | ? |
|  | Waters et al., 2004 | ? | ? | + | + | ? | + |
|  | Ondo et al., 2007 | + | + | ? | ? | – | + |
| Nicotine patch | Villafane et al., 2018 | + | + | – | + | + | + |
| Terguride | Pacchetti et al., 1993 | ? | ? | ? | ? | + | + |
| Perampanel | Eggert et al., 2010 | ? | – | + | + | ? | + |
|  | Lees et al., 2012 | + | ? | + | + | + | + |

**Supplementary Table 3.** Risk of bias assessment of included studies on surgical and experimental treatments for motor fluctuations in Parkinson's disease.

| Procedure | References | Random sequence generation | Allocation concealment | Blinding participants and personnel | Blinding outcome assessment | Incomplete outcome data | Selective reporting |
| --- | --- | --- | --- | --- | --- | --- | --- |
| GPi Deep Brain Stimulation DBS | Anderson et al., 2005 | ? | ? | – | + | + | + |
|  | Weaver et al., 2009 | + | ? | – | + | + | + |
|  | Follett et al., 2010 | + | + | + | + | + | + |
|  | Williams et al., 2010 | + | + | – | – | + | + |
|  | Weaver et al., 2012 | + | + | + | + | ? | + |
|  | Odekerken et al., 2013 | + | + | + | + | + | + |
|  | Sidiropoulos et al., 2016 | + | + | – | + | + | + |
| Pallidotomy unilateral | de Bie et al., 1999 | + | + | – | + | + | + |
|  | Vitek et al., 2003 | ? | ? | – | + | + | + |
|  | Esselink et al., 2004 | + | + | – | + | + | + |
|  | Esselink et al., 2006 | + | + | – | + | + | + |
|  | Coban et al., 2009 | ? | ? | ? | + | + | + |
| Subthalamotomy | Merello et al., 2008 | + | ? | – | – | + | + |
|  | Coban et al., 2009 | ? | ? | ? | + | + | + |
| Zona incerta DBS | Blomstedt et al., 2018 | + | ? | – | ? | – | ? |
| Glial Cell-Derived Neurotrophic Factor GDNF | Whone et al., 2019a | + | + | + | + | + | + |
|  | Whone et al., 2019b | + | + | + | + | + | + |

# GRADE assessment

**Supplementary Table 4.** GRADE Summary of Findings

| **Outcome** | **No. of participants (studies)** | **Relative effect** | **Absolute effect** | **Certainty of evidence (GRADE)** | **Comments** |
| --- | --- | --- | --- | --- | --- |
| **IPX066 (extended-release)** | | | | | |
| **OFF-time reduction (hours/day)** | 393 (1 RCT) | — | Mean difference −1.2 h (95% CI not reported) favouring IPX066 | ⊕⊕⊕⊕ High | IPX066 produced a clinically meaningful reduction of ≈1.2 h/day of OFF-time compared with immediate-release carbidopa/levodopa |
| **Functional improvement (UPDRS-II, ON-state)** | 393 (1 RCT) | — | Mean difference −0.9 points (P < 0.01) favoring IPX066, below the MCID (3 points) | ⊕⊕⊕⊕ High | Statistically significant but not clinically relevant improvement in activities of daily living |
| **Health-related quality of life impact (PDQ-39)** | 393 (1 RCT) | — | Mean difference −2.5 points (P < 0.05) favoring IPX066 – below the MCID (5 points) | ⊕⊕⊕⊕ High | Improvement on PDQ-39 did not reach clinical relevance; EQ-5D and SF-36 showed no significant differences between groups |
| The single high-quality phase III RCT supports that IPX066 (extended-release carbidopa-levodopa) yields a clinically meaningful reduction in OFF-time, but no important gains in function (UPDRS-II) or quality of life (PDQ-39) compared with immediate-release formulations. | | | | | |
| **Opicapone** | | | | | |
| **OFF-time reduction (hours/day)** | 1,464 (3 RCTs) | — | Mean difference ranged from ≈−0.7 to −2.0 h/day (favouring opicapone) | ⊕⊕⊕⊕ High | Two trials showed ≈120 min/day less OFF-time with corresponding ↑ ≈60 min/day more ON-time; a third trial showed a smaller benefit (~0.7 h/day). |
| **Functional improvement (UPDRS-II)** | 1,027 (2 RCTs) | — | One trial: no significant improvement; another: ~−1 point (off-med state), below the 3-point MCID | ⊕⊕⊕⊕ High | Effects on activities of daily living were statistically small and not clinically meaningful. |
| **Health-related quality of life impact (PDQ-39)** | 1,464 (3 RCTs) | — | No important difference vs placebo | ⊕⊕⊕⊕ High | Across trials, PDQ-39 did not show clinically meaningful improvement; EQ-5D (when assessed) was likewise not different. |
| Opicapone was judged "efficacious" for reducing OFF-time versus placebo; ON-time increased by ~60 min in two trials. Disability (UPDRS-II) changes were absent or minimal (~1 point, not clinically relevant), and HR-QoL (PDQ-39) showed no significant/clinically important improvement. | | | | | |
| **Pramipexole IR/ER** | | | | | |
| **OFF-time reduction (hours/day)** | 1,906 (6 RCTs) | — | Clinically meaningful reduction (≈1–2 h/day) favouring pramipexole | ⊕⊕⊕⊕ High | Multiple trials showed statistically significant and clinically relevant reductions in OFF time vs placebo for both IR and ER formulations. |
| **Functional improvement (MDS-UPDRS-II)** | 1,906 (6 RCTs) | — | Mean improvement of 3–5 points in the off-medication state | ⊕⊕⊕⊕ High | Significant improvements in activities of daily living (UPDRS-II) vs placebo, consistent across trials. |
| **Health-related quality of life impact (PDQ-39 / EQ-5D)** | 1,191 (4 RCTs) | — | Small improvement (~2–5 points), below the 5-point MCID | ⊕⊕⊕⊕ High | One study showed a clinically meaningful improvement on the PDQ-39; others found modest or no differences vs placebo. |
| Across several high-quality RCTs, pramipexole (IR and ER) demonstrated clinically meaningful reductions in OFF-time and improvements in functional capacity (UPDRS-II) in patients with Parkinson's disease experiencing motor fluctuations. Effects on health-related quality of life were modest and often below the threshold for clinical relevance. | | | | | |
| **Rotigotine** | | | | | |
| **OFF-time reduction (h/day)** | 2,311 (6 RCTs) | — | −0.9 to −1.8 h/day vs placebo across trials | ⊕⊕⊕⊕ High | Six RCTs consistently showed clinically meaningful reductions in OFF time; five reported a difference of>1 h/day. |
| **Functional improvement (UPDRS-II)** | 2,311 (6 RCTs) | — | Small-to-moderate improvement vs placebo | ⊕⊕⊕⊕ High | All six studies collected disability measures; four reported statistically significant improvements; two (incl. a large trial) were not significant. |
| **HR-QoL impact (PDQ-39)** | 351 (1 RCT) | — | Modest improvement; significance mixed across studies overall | ⊕⊕⊕◯ Moderate | Of the rotigotine RCTs, PDQ-39 was assessed in one pivotal study (plus PDQ-8 in another); only one study showed a statistically significant QoL gain. |
| Across six double-blind RCTs totalling ~2.3k participants, rotigotine patches as an adjunct to levodopa reduce diary-based OFF-time by ~0.9–1.8 hours/day, with high-certainty evidence. Disability (UPDRS-II) generally improves in parallel (significant in four studies), supporting high certainty for functional benefit. HR-QoL effects are modest and inconsistently significant; given that only a subset of trials assessed PDQ-39/PDQ-8 and results were mixed, the certainty of the QoL evidence is rated moderate here. | | | | | |
| **Safinamide** | | | | | |
| **OFF-time reduction (h/day)** | 1,931 (4 RCTs) | — | ≈ −1.0 h/day vs placebo (and ≈ +1.0 h/day ON-time without troublesome dyskinesia) | ⊕⊕⊕⊕ High | All four RCTs collected diary outcomes and consistently showed a clinically meaningful reduction in OFF time (~1 h) and an increase in dyskinesia-free ON time. |
| **Functional improvement (UPDRS-II)** | 1,931 (4 RCTs) | — | Small improvement vs placebo (directionally favourable across trials) | ⊕⊕⊕⊕ High | UPDRS-II (ON) was a prespecified endpoint in all four trials. Effects were generally in favour of safinamide. |
| **HR-QoL impact (PDQ-39)** | 1,262 (3 RCTs) | — | Modest, mixed results | ⊕⊕⊕◯ Moderate | PDQ-39 was assessed in Schapira 2017, Hattori 2020b, and Wei 2022; findings varied, with significant benefit in only a subset of studies. |
| Across four high-quality RCTs (n=1,931), safinamide as an adjunct to levodopa reduces diary-based OFF-time by about 1 hour/day and increases ON-time without troublesome dyskinesia by about 1 hour/day (high certainty). Functional ability (UPDRS-II) shows small improvements consistent with symptomatic benefit (high certainty). Quality-of-life effects (PDQ-39) are modest and inconsistent across studies (moderate certainty). | | | | | |
| **Levodopa–carbidopa intestinal gel** | | | | | |
| **OFF-time reduction (hours/day)** | 141 (2 RCTs) | — | Mean reduction −1.9 to −2.3 h/day vs optimised oral therapy | ⊕⊕⊕◯ Moderate | Both studies demonstrated significant reductions in daily OFF time compared with oral levodopa. Olanow 2014: −1.9 h/day; Chung 2022: −2.3 h/day. Consistent benefit observed across trials. |
| **Functional improvement (UPDRS-II)** | 141 (2 RCTs) | — | Mean difference −3.0 to −4.5 points vs oral therapy | ⊕⊕⊕◯ Moderate | Both RCTs reported significant improvement in UPDRS-II (ON-state) vs oral therapy, indicating better activities of daily living. Effect magnitude is clinically meaningful. |
| **Health-related quality of life impact (PDQ-39)** | 141 (2 RCTs) | — | Mean improvement −6 to −9 points vs oral therapy | ⊕⊕⊕◯ Moderate | LCIG significantly improved HR-QoL (PDQ-39 summary index) beyond the 5-point MCID in both RCTs, reflecting tangible patient benefit. |
| Evidence from two double-blind RCTs (total n = 141) shows that levodopa–carbidopa intestinal gel (LCIG) provides clinically meaningful reductions in daily OFF-time (≈2 h/day) and improvements in functional ability (UPDRS-II ≈ 3–5 points) and quality of life (PDQ-39 ≈ 6–9 points) compared with optimised oral levodopa. The effects are consistent and of clear clinical relevance, but the overall certainty is rated moderate due to small sample sizes, short duration (12–24 weeks), and the invasive mode of administration. LCIG is classified as efficacious for treating advanced PD with disabling fluctuations, though its use is best reserved for patients unresponsive to optimised oral or transdermal therapy. | | | | | |
| **Foslevodopa–foscarbidopa subcutaneous** | | | | | |
| **OFF-time reduction (h/day)** | 174 (1 RCT) Studies_list | — | −1.8 h/day vs oral levodopa (P=0.002) | ⊕⊕⊕◯ Moderate | Primary diary outcomes showed ↑ ON-time without troublesome dyskinesia by ~1.8 h/day (P<0.01) in the infusion group. |
| **Functional improvement (MDS-UPDRS II)** | 174 (1 RCT) Studies_list | — | No important difference (MD not significant; exact value not reported in summary) | ⊕⊕⊕◯ Moderate | MDS-UPDRS II was prespecified and collected, but evidence on disability is insufficient for a benefit statement. |
| **HR-QoL impact (PDQ-39)** | 174 (1 RCT) Studies_list | — | No significant difference (MD not reported) | ⊕⊕⊕◯ Moderate | PDQ-39 and EQ-5D-5L were collected; evidence on HR-QoL is insufficient to show benefit. |
| In a single, double-blind, double-dummy phase 3 RCT (n=174; 12 weeks), continuous subcutaneous foslevodopa–foscarbidopa produced a clinically meaningful reduction in diary-based OFF-time (≈1.8 h/day) and a parallel increase in ON-time without troublesome dyskinesia (≈1.8 h/day) versus optimised oral levodopa. Effects on activities of daily living (MDS-UPDRS II) and quality of life (PDQ-39/EQ-5D-5L) were not demonstrably improved, and the available summary provides insufficient evidence for these outcomes. Overall certainty is rated moderate. | | | | | |
| **Apomorphine infusion** | | | | | |
| **OFF-time reduction (hours/day)** | 246 (3 RCTs) | — | Mean reduction ≈ −1.8 h/day vs placebo/oral levodopa | ⊕⊕⊕◯ Moderate | All three RCTs demonstrated significant, clinically relevant reductions in OFF time. TOLEDO (n=106) was the pivotal trial (−2.5 h/day). Hattori 2014 and Olanow 2020 also showed OFF reduction trends with different formulations (infusion and sublingual, respectively). |
| **Functional improvement (UPDRS-II)** | 140 (2 RCTs) | — | Small to moderate improvement (−2 to −4 points) | ⊕⊕⊕◯ Moderate | UPDRS-II was assessed in Hattori (2014) and Olanow (2020), and apomorphine improved it slightly compared with placebo or oral levodopa; effects were consistent but modest. |
| **Health-related quality of life impact (PDQ-8 / PDQ-39)** | 246 (3 RCTs) | — | Modest improvement (≈ −3 to −5 points) | ⊕⊕⊕◯ Moderate | PDQ-8 improved in TOLEDO; Olanow 2020 (sublingual) and Hattori 2014 (Japanese cohort) reported no or minor change. The overall HR-QoL effect is favourable but not consistently clinically meaningful. |
| Across three RCTs (total n ≈ 246), apomorphine, administered either as continuous subcutaneous infusion or sublingual film, significantly reduced daily OFF-time by ~2 hours/day and increased ON-time without troublesome dyskinesia, compared with placebo or optimised oral therapy. Functional improvement (UPDRS-II) was not evaluated in TOLEDO, but in the other two trials, it showed modest (2–4-point) improvements, consistent with better daily functioning. Quality of life (PDQ-8/PDQ-39) improved slightly, driven mainly by TOLEDO's PDQ-8 results, though not always reaching clinical significance. Overall, the evidence is moderate in certainty, which classifies apomorphine infusion as efficacious for motor fluctuations, but is based on a limited number of RCTs and heterogeneous outcome reporting. | | | | | |
| **Rasagiline** | | | | | |
| **OFF-time reduction (h/day)** | 2,131 (5 RCTs) | — | ≈ −0.5 to −0.9 h/day vs placebo | ⊕⊕⊕◯ Moderate | All five RCTs reported diary OFF-time as the primary outcome; pooled direction favours rasagiline, but the average benefit is not clinically meaningful per MDS review. |
| **Functional improvement (UPDRS-II)** | 1,807 (4 RCTs) | — | ≈ −1.0 to −1.7 points vs placebo | ⊕⊕⊕◯ Moderate | Four RCTs reported small, statistically significant but not clinically meaningful improvements in disability (UPDRS-II). |
| **HR-QoL impact (PDQ-39)** | 1,483 (3 RCTs) | — | Statistically significant but < MCID | ⊕⊕⊕◯ Moderate | Three trials assessed PDQ-39; improvements were statistically significant but below clinical relevance thresholds. |
| Across five randomised, double-blind trials (n≈2,131), rasagiline as an add-on to levodopa reduces diary OFF-time by ~0.5–0.9 hours/day, but this average reduction is not clinically meaningful. Disability (UPDRS-II) improves by ~1–2 points, also below standard MCIDs; PDQ-39 shows statistical gains that do not reach clinical relevance. Overall certainty is moderate, judging rasagiline likely efficacious but with modest magnitudes of benefit on patient-important outcomes. | | | | | |
| **Zonisamide** | | | | | |
| **OFF-time reduction (h/day)** | 722 (2 RCTs) | — | ≈ −0.7 to −1.4 h/day (−40 to −85 min) vs placebo | ⊕⊕⊕◯ Moderate | Dose-response signal across 25–100 mg; diaries collected; pooled direction favours zonisamide. |
| **Functional improvement (UPDRS-II)** | 375 (1 RCT) | — | ≈ −1 point (statistically but not clinically significant) | ⊕⊕⊕◯ Moderate | Disability was assessed in one study; small effect below MCID. |
| **HR-QoL impact (PDQ-39/EQ-5D)** | Not assessed in eligible trials | — | — | — | Not assessed in the zonisamide RCTs included in the review. |
| Across two Japanese multicenter, double-blind RCTs (total n=722), zonisamide (25–100 mg once daily) as an adjunct to levodopa reduced daily OFF-time by ~40–85 minutes/day with a dose-responsive pattern (moderate certainty). Motor impairment on-med (UPDRS-III) improved modestly, and disability (UPDRS-II) was formally assessed in one trial, showing a ~1-point improvement—statistically significant but not clinically meaningful (moderate certainty). Health-related quality of life was not assessed in the eligible trials. Overall, zonisamide is likely efficacious for treating motor fluctuations, supported by moderate-quality evidence. | | | | | |
| **Ropinirole** | | | | | |
| **OFF-time reduction (hours/day)** | 4,466 (14 RCTs) | — | Mean difference ≈ −0.8 to −1.2 h/day vs placebo | ⊕⊕⊕◯ Moderate | All 14 RCTs included diary-based OFF-time; the pooled direction favours ropinirole, with borderline clinical relevance. Immediate-release (IR) studies are consistently significant; prolonged-release (PR) and patch formulations show comparable effects but with some risk-of-bias limitations. |
| **Functional improvement (UPDRS-II)** | 4,466 (14 RCTs) | — | Mean difference ≈ −1 to −2 points vs placebo | ⊕⊕⊕◯ Moderate | UPDRS-II (ADL) improved slightly across trials but did not reach the minimal clinically important difference (MCID ≈ 3 points). Effects were consistent between IR and PR formulations, and stable over 12–24 weeks. |
| **Health-related quality of life (PDQ-39 / EQ-5D)** | 3,271 (9 RCTs) | — | Small, non–clinically relevant improvement (≈ −3 to −4 PDQ-39 points) | ⊕⊕⊕◯ Moderate | Nine RCTs reported HR-QoL measures (mainly PDQ-39). Most found modest improvements, generally below the MCID (5 points)—no significant differences between IR and PR formulations. |
| Across 14 randomised controlled trials (n ≈ 4,466), ropinirole (immediate- and prolonged-release formulations) as an adjunct to levodopa reduces OFF-time by about 1 hour/day and provides small, statistically significant but not clinically meaningful improvements in activities of daily living (UPDRS-II) and quality of life (PDQ-39 / EQ-5D). Immediate-release formulations show the most consistent results, while prolonged-release formulations yield comparable effects with a slightly higher risk of bias in some studies. Overall certainty is rated moderate, classifying ropinirole as efficacious (IR) and likely efficacious (PR) for reducing motor fluctuations, though with limited functional or QoL impact. | | | | | |
| **Entacapone** | | | | | |
| **OFF-time reduction (hours/day)** | 1,570 (7 studies) | — | Reduction ≈ 0.5–1.0 h/day vs control | ⊕⊕⊕◯ Moderate | Consistent diary-based improvements across trials show that entacapone significantly reduces daily OFF-time compared with placebo, typically by 30–60 minutes. Benefits are modest but clinically relevant for some patients, reflecting the drug's role in extending levodopa bioavailability and stabilising motor response. |
| **Functional improvement (UPDRS-II)** | 1,570 (7 studies) | — | Small improvement (≈ –1 point), not clinically important | ⊕⊕⊕◯ Moderate | UPDRS-II improved slightly, paralleling the reduction in OFF time, but the average change did not reach the minimal clinically important difference (≈ 3 points). The effect reflects a mild improvement in daily functioning, largely mediated by a smoother levodopa response rather than direct antiparkinsonian action. |
| **Health-related quality of life (PDQ-39 / SF-36 / EQ-5D)** | 952 (4 studies) | — | Modest/neutral effect; generally < PDQ-39 MCID | ⊕⊕⊕◯ Moderate | Quality-of-life outcomes showed a trend toward better well-being (e.g., PDQ-39 improvement of ≈ 2–4 points) but below the 5-point clinical threshold. Patients reported fewer daily fluctuations but no substantial overall gains in life quality, indicating symptomatic relief without functional transformation. |
| Across eight randomised studies of entacapone added to levodopa (n≈1,570 for motor/ADL outcomes; n≈952 for QoL), evidence shows a consistent reduction in daily OFF-time of roughly 0.5–1.0 hours and a small improvement in UPDRS-II that generally does not reach clinical importance. Quality-of-life effects (PDQ-39/SF-36/EQ-5D) are modest or neutral overall. This profile aligns with contemporary evidence syntheses that consider entacapone efficacious for smoothing levodopa response, with moderate-quality certainty due to heterogeneity and limited clinically meaningful gains on disability/QoL despite clear diary benefits. | | | | | |
| **Amantadine** | | | | | |
| **OFF-time reduction (h/day)** | 259 (3 RCTs) | — | Directionally favours amantadine (secondary endpoint in ER trials); diaries show ↓ OFF and ↑ ON-time without troublesome dyskinesia. | ⊕⊕⊕◯ Moderate | Two phase-3 ER trials (EASE LID 3; EASE LID) consistently improved diary measures alongside dyskinesia reduction; the withdrawal trial (AMANDYSK) corroborates the benefit by showing OFF/ON pattern worsening after stopping amantadine. |
| **Functional improvement (UPDRS-II)** | 203 (2 RCTs) | — | Small to neutral change (MDS-UPDRS collected; disability effects limited). | ⊕⊕⊕◯ Moderate | Trials were powered for dyskinesia; UPDRS-II effects, when reported, are modest and below typical MCIDs—benefit is mainly via better ON-time quality. |
| **Health-related quality of life (PDQ-39 / EQ-5D)** | Not assessed in the three included trials | — | — | — | QoL instruments were not reported in these specific studies. |
| Across three randomised trials (n = 259 randomised: AMANDYSK n=56; EASE LID 3 n=77; EASE LID n=126), amantadine (particularly ADS-5102 extended-release) shows consistent diary benefits—reduced OFF-time and increased ON-time without troublesome dyskinesia—while disability (UPDRS-II) changes are small/neutral, and HR-QoL was not reported in these studies. Overall certainty is moderate, given the secondary-endpoint status of OFF-time in ER trials and limited disability/QoL data. | | | | | |
| **Istradefylline** | | | | | |
| **OFF-time reduction (h/day)** | 2,583 (8 RCTs) | — | Consistently favours istradefylline (↓ daily OFF; ↑ ON time)—primary endpoint in multiple trials using home diaries. | ⊕⊕⊕◯ Moderate | Pooled across large, multicenter RCTs in North America, Europe, and Japan (e.g., Hauser 2008 n=231; LeWitt 2008 n=195; Stacy 2008 n=395; Mizuno 2010 n=363; Pourcher 2012 ITT n=584; Mizuno 2013 n=373). Effect measured with standardised 24-h PD diaries. |
| **Functional improvement (UPDRS-II)** | 373 (1 RCT) | — | Small change; disability effects are limited relative to OFF-time benefits. | ⊕⊕⊕◯ Moderate | UPDRS subscales were variably reported; at least one Japanese trial explicitly collected UPDRS I–IV (includes II). Other trials emphasised UPDRS III/total rather than II specifically. |
| **Health-related quality of life (HR-QoL: PDQ-39 / EQ-5D)** | 584 (1 RCT) | — | Directionally favourable but modest; QoL assessed in the large North American dose-response trial. | ⊕⊕⊕◯ Moderate | QoL was prespecified in KW-6002-US-018; most other trials focused on OFF/ON diaries and motor scores. |
| Across eight trials, istradefylline consistently reduces daily OFF-time when added to levodopa, with large, multicenter RCTs providing the bulk of the evidence (aggregated n≈2,141 for OFF-time, where participant counts are available in your file). Reporting of UPDRS-II is limited (explicit in at least one RCT), and HR-QoL was assessed in the large North American dose-response study (ITT n=584). Overall, the balance of evidence supports moderate-certainty confidence in OFF-time benefits, with more minor or inconsistently reported effects on disability and QoL. | | | | | |
| **Levodopa–carbidopa CR** | | | | | |
| **OFF-time reduction (h/day)** | 254 (2 RCTs) | — | Direction favours CR in one study; null in another | ⊕⊕◯◯ Low | Two trials recorded diary ON/OFF (1992, 1996). The 1996 multicenter RCT prespecified ON/OFF as primary outcomes (n=170), and the 1992 RCT included diaries among outcomes (n=84), but effects were small/inconsistent, and the methods were dated (nonstandard scales, incomplete reporting). |
| **Functional improvement (UPDRS-II)** | 0 (0 RCTs) | — | Not reported as UPDRS-II | — | Functional scales used were NYUPDRS/NWUDS rather than UPDRS-II. |
| **HR-QoL impact (PDQ-39 / EQ-5D)** | 0 (0 RCTs) | — | Not assessed | — | None of the included CR trials reported modern HR-QoL instruments (PDQ-39/EQ-5D). The evidence is therefore insufficient for QoL. |
| Across three historical trials, levodopa–carbidopa CR shows inconsistent, small effects on diary OFF-time compared with IR or placebo, with no demonstrated improvements in disability and no HR-QoL data using modern instruments. The evidence base is limited by older trial designs, nonstandard functional scales (NYUPDRS/NWUDS instead of UPDRS-II), and sparse reporting. The overall conclusion is that there is insufficient evidence for the efficacy of LC-CR in treating motor fluctuations (and no proven benefit on disability or QoL). | | | | | |
| **Selegiline** | | | | | |
| **OFF-time reduction (h/day)** | 328 (4 RCTs) | — | Mixed/mostly small; only one trial showed a ≥1-h decrease | ⊕⊕◯◯ Low | OFF diaries were collected in Heinonen 1989 (n=19), Hubble 1993 (n=19), Waters 2004 (n=140), and Ondo 2007 (n=150); overall results were heterogeneous and generally modest, with just one clinically meaningful result. |
| **Functional improvement (UPDRS-II)** | 290 (2 RCTs) | — | Small/not clinically important | ⊕⊕◯◯ Low | UPDRS-II (ADL) was reported in Waters 2004 (n=140) and Ondo 2007 (n=150); changes were modest and below MCID. Earlier crossover trials used nonstandard disability scales (not UPDRS-II). |
| **HR-QoL impact (PDQ-39 / EQ-5D)** | 0 (0 RCTs) | — | Not assessed in these trials | — | None of the five selegiline fluctuation RCTs in scope reported PDQ-39/EQ-5D; evidence is therefore insufficient for QoL. |
| Across five RCTs, the overall evidence for selegiline to treat motor fluctuations is insufficient/low quality. Four trials collected OFF-time diaries, but only one achieved a clinically meaningful reduction (≥1 h/day). Disability benefits on the UPDRS-II were small and not clinically important, and no study reported the use of modern HR-QoL instruments. Limitations include small sample sizes, crossover designs, nonstandard disability measures in early trials, and heterogeneous results. In agreement with Bie et al. (2025), we rate the certainty ⊕⊕◯◯ as Low () and conclude that selegiline lacks sufficient evidence for meaningful improvement in OFF-time, daily function, or QoL in fluctuating PD. | | | | | |
| **Nicotine patch** | | | | | |
| OFF-time reduction (hours/day) | 67 (1 RCT) | — | No significant change vs placebo | ⊕⊕◯◯ Low | A 6-month, double-blind RCT (n=67) compared transdermal nicotine (up to 14 mg/day) with placebo patches. Diaries and UPDRS assessments showed no difference in OFF or ON time between groups. |
| Functional improvement (UPDRS-II) | 67 (1 RCT) | — | No improvement in UPDRS-II vs placebo | ⊕⊕◯◯ Low | UPDRS-II and III scores did not differ between nicotine and placebo groups after 6 months; changes were small and not clinically meaningful. |
| Health-related quality of life (PDQ-39) | 67 (1 RCT) | — | No difference vs placebo | ⊕⊕◯◯ Low | PDQ-39 total and domain scores showed no significant improvement. Mild skin reactions and nausea were common adverse events. |
| The only available RCT (n = 67) assessed nicotine patches (up to 14 mg/day) versus placebo in PD patients with motor fluctuations over 6 months. The trial found no significant differences in OFF-time, UPDRS-II, or PDQ-39 outcomes between treatment groups. Although nicotine was generally well tolerated, the evidence is limited to a single, small study with no replication and modest methodological quality. The overall evidence for nicotine as an antiparkinsonian therapy is considered insufficient. | | | | | |
| **Terguride** | | | | | |
| OFF-time reduction (hours/day) | 12 (1 RCT) | — | Trend toward OFF-time reduction, not statistically significant | ⊕⊕◯◯ Low | Small crossover RCT (n=12) comparing terguride (partial dopamine D₂ agonist) vs placebo as add-on to levodopa. Diary OFF-time and clinical ratings improved slightly in some participants, but variability was high and results not significant. |
| Functional improvement (UPDRS-II or equivalent) | 12 (1 RCT) | — | No clear improvement | ⊕⊕◯◯ Low | Functional outcomes based on total and subscore analysis of UPDRS showed minor, non-significant changes. Study underpowered and used older disability scales. |
| Health-related quality of life (PDQ-39 / EQ-5D) | 0 (0 RCTs) | — | Not assessed | — | QoL instruments were not used; only motor and clinical scores were collected. |
| The only available RCT (n = 12) investigated terguride, a mixed dopamine D₂ receptor agonist–antagonist, as an add-on to levodopa in fluctuating PD. The trial reported a non-significant trend toward reduced OFF-time and minor, clinically irrelevant changes in motor and functional scores. No health-related quality-of-life measures were included. Evidence is limited to a single, very small study with a short duration, insufficient statistical power, and the use of older scales that are not comparable to modern UPDRS-II or PDQ-39. Accordingly, the overall certainty is rated low, reflecting insufficient evidence to support the efficacy of terguride in reducing motor fluctuations or improving daily functioning. | | | | | |
| **Perampanel** | | | | | |
| **OFF-time reduction (hours/day)** | 757 (2 RCTs) | — | No significant difference vs placebo | ⊕⊕◯◯ Low | Both RCTs evaluated perampanel (an AMPA receptor antagonist) as an add-on to levodopa. Neither study demonstrated a significant reduction in daily OFF-time. The larger phase 3 trial (Lees 2012, n=549) failed to meet its primary endpoint. Eggert 2010, n=208) also found no consistent diary improvement. |
| **Functional improvement (UPDRS-II)** | 757 (2 RCTs) | — | No significant change | ⊕⊕◯◯ Low | UPDRS-II and III were collected as secondary endpoints in both trials. No statistically or clinically meaningful differences were detected between groups. Effects were directionally neutral. |
| **Health-related quality of life (PDQ-39 / EQ-5D)** | 549 (1 RCT) | — | No difference vs placebo | ⊕⊕◯◯ Low | The large phase 3 RCT (Lees 2012) included PDQ-39, showing no improvement in overall or domain scores. No HR-QoL data were reported in Eggert 2010. |
| Evidence from two double-blind, randomised trials (total n = 757) shows no significant benefit of perampanel for reducing OFF-time, improving activities of daily living (UPDRS-II), or enhancing quality of life (PDQ-39). Both studies were well-conducted phase 2/3 trials, but consistently negative across outcomes. Perampanel was well tolerated but did not demonstrate clinically relevant efficacy. The overall certainty of evidence is low due to a limited number of trials, modest sample sizes, and uniformly null results. | | | | | |
| **GPi Deep Brain Stimulation DBS** | | | | | |
| **OFF-time reduction / ON-time (h/day)** | 1,230 (6 RCTs) | — | ON-time without troublesome dyskinesia ↑ ≈4–5 h/day with DBS vs best medical therapy (BMT). | ⊕⊕⊕⊕ High | Magnitude driven by large multicenter RCTs; DBS (both targets) markedly increased good ON-time vs BMT. For GPi specifically, RCTs show benefits comparable to STN. |
| **Functional improvement (UPDRS-II)** | 864 (5 RCTs) | — | UPDRS-II ↓ ≈4–5 points with DBS vs BMT; GPi ≈ STN at 24 mo. | ⊕⊕⊕⊕ High | Disability improved meaningfully in randomised comparisons; GPi and STN yielded similar ADL gains at 24 months and remained stable at 36 months. |
| **Health-related quality of life (PDQ-39)** | 920 (3 RCTs) | — | PDQ-39 total ↓ ≈12 points with DBS vs BMT; GPi shows trends comparable to STN. | ⊕⊕⊕⊕ High | QoL improvements large and consistent in trials in which PDQ-39 was primary/secondary. (NSTAPS used PDQL and is not counted in this PDQ-39 total.) |
| Across randomised trials, GPi DBS delivers large, clinically meaningful benefits for patients with motor fluctuations: substantial gains in good ON-time (~4–5 h/day), meaningful ADL improvement (UPDRS-II ~4–5 points), and robust QoL improvements (PDQ-39 ~12 points) when compared with best medical therapy. Head-to-head target trials show GPi ≈ STN for motor and functional outcomes at 24–36 months, with some favourable trends for GPi in the dyskinesia and QoL domains in secondary analyses. | | | | | |
| **Pallidotomy unilateral** | | | | | |
| **OFF-time reduction (h/day)** | 37 (1 RCT) | — | Direction favours pallidotomy in the single diary-based trial | ⊕⊕⊕◯ Moderate | Only one RCT systematically assessed OFF/ON time with diaries (de Bie 1999, n=37); other trials emphasised UPDRS and dyskinesia rather than time-in-state, so evidence for OFF-time is limited in breadth. |
| **Functional improvement (UPDRS-II)** | 117 (4 RCTs) | — | Clinically meaningful disability improvements vs control at ~6 months | ⊕⊕⊕◯ Moderate | Four trials reported ADL/disability: de Bie 1999 (UPDRS-II listed), Vitek 2003 (UPDRS I–IV prespecified), Esselink 2004 and Esselink 2006 (UPDRS-II listed). Across trials, UPDRS-II and Schwab & England improved with pallidotomy vs medical therapy; the magnitude judged clinically important in the two medical-therapy comparators. |
| **HR-QoL impact (PDQL)** | 71 (2 RCTs) | — | Improvement from baseline; between-group differences favour pallidotomy vs medical therapy | ⊕⊕⊕◯ Moderate | QoL instruments used were PDQL (not PDQ-39). Reported in de Bie 1999 and Esselink 2004; the 12-month Esselink 2006 paper is a follow-up of the same cohort and not double-counted. |
| Across five randomised studies, unilateral pallidotomy shows meaningful improvements in disability (UPDRS-II/Schwab & England) and QoL (PDQL) versus medical therapy, with limited, single-trial evidence suggesting reduced OFF-time. Head-to-head comparisons indicate STN-DBS outperforms unilateral pallidotomy for off-medication motor impairment, while a very small trial found pallidotomy ≈ subthalamotomy on clinical scales. Overall, the body of evidence is moderate in certainty due to small sample sizes, lack of sham control, and reliance on older measures/short follow-up in some trials. Yet, it consistently supports functional gains with pallidotomy in appropriately selected patients. | | | | | |
| **Subthalamotomy** | | | | | |
| **OFF-time reduction (h/day)** | 0 (0 RCTs) | — | Not assessed in these trials | ⊕◯◯◯ Very Low | Neither RCT collected modern home-diary OFF/ON-time as an outcome; both focused on UPDRS and dyskinesia/clinical scales. |
| **Functional improvement (UPDRS-II)** | 26 (2 RCTs) | — | Small–moderate improvement vs comparators (direction favours subthalamotomy), clinical importance uncertain | ⊕⊕◯◯ Low | Both trials recorded UPDRS subscores, including ADL (Part II): Merello 2008 (3-arm pilot: bilateral subthalamotomy vs STN-DBS vs combined; 16 randomised, 1 death) and Coban 2009 (pallidotomy vs subthalamotomy; n=10). Small samples, heterogeneous comparators, and pilot design limit confidence. |
| **Health-related quality of life (PDQ-39 / EQ-5D)** | 0 (0 RCTs) | — | Not assessed | — | Neither Merello 2008 nor Coban 2009 reported PDQ-39 or EQ-5D; the evidence of outcomes is absent. |
| Evidence for subthalamotomy in fluctuating PD is limited and methodologically heterogeneous. Across two small RCTs (n = 26 total randomised for ADL outcomes), UPDRS-based disability measures tended to improve after subthalamotomy relative to surgical comparators, but no trials reported diary-based OFF-time or HR-QoL (PDQ-39/EQ-5D). Designs (pilot/three-arm surgical comparison; lesion-vs-lesion randomisation), very small samples, and lack of standardised patient-important outcomes constrain certainty. We rate the overall certainty as low (insufficient evidence) for reducing OFF-time, improving daily function to a clinically meaningful extent, or enhancing quality of life. | | | | | |
| **Zona incerta DBS** | | | | | |
| OFF-time reduction (h/day) | 0 (0 RCTs) | — | Not assessed | — | The RCT did not include home diary OFF/ON time; the primary outcomes were UPDRS-III and PDQ-39. |
| Functional improvement (UPDRS-II) | 0 (0 RCTs) | — | Not assessed | — | UPDRS-II was not prespecified/reported in the trial; focus was UPDRS-III (OFF) and QoL. |
| Health-related quality of life (PDQ-39) | 19 (1 RCT) | — | Improved from baseline in both groups; no between-group difference | ⊕⊕◯◯ Low | At 6 months, 19 participants completed evaluations (9 DBS, 10 BMT). PDQ-39 improved from baseline in both arms, without significant between-group differences; assessor-blinded only, not patient-blinded. |
| Evidence for zona incerta DBS in fluctuating PD is limited to one small, single-centre RCT with assessor-only blinding and no diary-based OFF/ON outcomes. At 6 months, motor impairment (UPDRS-III OFF) improved within the DBS arm, but QoL (PDQ-39) improved similarly from baseline in both DBS and BMT groups, yielding no significant between-group difference. With no UPDRS-II or OFF-time data and only 19 completers, the overall certainty is low (insufficient evidence). | | | | | |
| **Glial Cell-Derived Neurotrophic Factor GDNF** | | | | | |
| **OFF-time reduction (h/day)** | 41 (1 RCT) | — | No clinically relevant difference vs placebo | ⊕⊕◯◯ Low | The single-centre, double-blind RCT collected PD home diary ratings but did not demonstrate a meaningful reduction in OFF time compared with placebo over 40 weeks. Outcomes (including diaries) are listed in the protocol summary of your file. |
| **Functional improvement (UPDRS-II)** | 41 (1 RCT) | — | No important change vs placebo | ⊕⊕◯◯ Low | UPDRS-II (ADL) was a prespecified secondary outcome; the RCT did not show a clinically relevant advantage of GDNF over placebo. (Primary endpoint was OFF-state UPDRS III and was negative.) |
| **Health-related quality of life (PDQ-39 or EQ-5D)** | 41 (1 RCT) | — | No difference vs placebo | ⊕⊕◯◯ Low | PDQ-39 and EQ-5D were collected as supplementary endpoints in the RCT; between-group differences were not clinically meaningful. |
| Evidence for intermittent intraputaminal GDNF consists of one small, single-centre RCT (n = 41) with a negative primary endpoint and no clinically meaningful advantages on OFF-time, ADL (UPDRS-II), or HR-QoL over 40 weeks; the open-label extension (all 41 participants) does not change this conclusion. Given the single RCT, surgical/device complexity, and lack of replication, certainty is low (insufficient evidence), concluding that current clinical evidence does not support a patient-important benefit of GDNF on fluctuations, function, or QoL. | | | | | |
